# Nociception related biomolecules in the adult human saliva: A scoping review with additional quantitative focus on cortisol

**DOI:** 10.1177/17448069241237121

**Published:** 2024-03-05

**Authors:** Roxaneh Zarnegar, Angeliki Vounta, Qiuyuan Li, Sara S Ghoreishizadeh

**Affiliations:** 1 Institute of Orthopaedics and Musculoskeletal Science, 4919 University College London, London, UK; 2 Royal National Orthopaedic Hospital NHS Trust, Stanmore, UK; 3 Department of Rehabilitation, 558113 Shenzhen University General Hospital, Shenzhen, China; 4 Department of Electronic and Electrical Engineering, 4919 University College London, London, UK

**Keywords:** Acute pain, biomarkers, experimental pain, induced pain, saliva, scoping review

## Abstract

Nociception related salivary biomolecules can be useful patients who are not able to self-report pain. We present the existing evidence on this topic using the PRISMA-ScR guidelines and a more focused analysis of cortisol change after cold pain induction using the direction of effect analysis combined with risk of bias analysis using ROBINS-I. Five data bases were searched systematically for articles on adults with acute pain secondary to disease, injury, or experimentally induced pain. Forty three articles met the inclusion criteria for the general review and 11 of these were included in the cortisol-cold pain analysis. Salivary melatonin, kallikreins, pro-inflammatory cytokines, soluable TNF-α receptor II, secretory IgA, testosterone, salivary α-amylase (sAA) and, most commonly, cortisol have been studied in relation to acute pain. There is greatest information about cortisol and sAA which both rise after cold pain when compared with other modalities. Where participants have been subjected to both pain and stress, stress is consistently a more reliable predictor of salivary biomarker change than pain. There remain considerable challenges in identifying biomarkers that can be used in clinical practice to guide the measurement of nociception and treatment of pain. Standardization of methodology and researchers’ greater awareness of the factors that affect salivary biomolecule concentrations are needed to improve our understanding of this field towards creating a clinically relevant body of evidence.

## Introduction

Effective pain management is a humanitarian responsibility and is essential to recovery and rehabilitation after surgery and trauma.^1^ Achieving it relies on robust methods for the assessment of pain and nociception. Pain is by nature subjective^2^ and acute pain assessment methods rely on self-reporting, using either scales (predominantly in acute pain) or questionnaires (predominantly in chronic pain). These methods are unhelpful when patients cannot self-report, for example, infants and young children, people under anaesthesia, or those with cognitive disabilities and mobility impairments. In these circumstances assessments based on behavioural and physiological indicators are used ^3,4^ which rely on the expertise of healthcare professionals, limiting their reliability.^5^ Further, they are not specific and may indicate other physiological or pathological processes.^6^ The relationship between pain and nociception (the level of activity in neuronal pathways after a noxious stimulus) is not straightforward and can particularly be affected by stress. Nonetheless when pain self-reporting cannot be used, a reliable assessment method based on nociception, such as monitoring the bio-fluid levels of molecules related to nociceptive signaling would enable clinicians to titrate analgesics more effectively. Saliva is a favorable bio-fluid because it can be obtained rapidly and non-invasively, when compared to, for example, blood or cerebrospinal fluid.

We aimed to collate the evidence on salivary nociception-related biomolecules in order to (1) identify potential biomarkers for acute pain, (2) determine whether change in biomolecule levels correlates with pain intensity and (3) whether this is different between the sexes. After article selection in line with inclusion criteria, it was evident that most of the studies in this field relate to change in salivary cortisol with experimentally induced cold pain and we have therefore done a more detailed review of this.

## Methods

### Design

We used the Preferred Reporting Items for Systematic Reviews and Meta-Analyses Extension for Scoping Reviews (PRISMA-ScR).^7^ The protocol was registered with the Open Science Framework.^8^ The review has one deviation from the registered protocol. This has been explained in the data synthesis section.

### Search strategy

A preliminary search was conducted in Medline to develop the key search items. A systematic literature search was done in Ovid MEDLINE, Ovid EMBASE, Web of Science, CENTRAL and PubMed in July 2020. There were no limitations by study design, language, or publication year. Email alerts were set up until 31st December 2022. The final search strategy is reported in the registered protocol.

### Study selection

Two reviewers removed duplicates and assessed titles and abstracts independently. The full texts of potentially relevant articles were screened against the inclusion and exclusion criteria (Table 1) and reasons for exclusion were recorded. Disagreements were resolved by consensus between all authors. The reference lists of included articles were hand-searched to identify additional relevant articles.

**Table 1.** Inclusion and exclusion criteria for selecting the sources of evidence.

Attribute	Inclusion criteria	Exclusion criteria
Study population	Human adults (≥18 years)	• Age <18 years
		• Animal studies
		• Studies with participants who
		- had chronic pain, a chronic painful condition, or acute pain that is an exacerbation or flare-up of a chronic or recurrent pain problem (for example migrainous acute headache)
		- had labour pain or postnatal pain
	- With experimentally induced acute pain on a background of no pain or a background of a chronic painful condition	- had a condition that disrupts the normal physiological conditions in the oral cavity (e.g. oral mucositis, oral diseases, or acute dental pain)
	- With acute pain conditions (such as post-operative pain, burns pain, zoster pain, acute (spinal) disc prolapse, fracture pain, renal colic, or biliary colic) of less than 6 weeks duration	- had substance dependence or abuse
Type of article	• Peer-reviewed published observational studies in any setting	Review articles, case reports, conference abstracts, publications without data, letters, and comments
	• Studies in any language and with any publication date	
Study outcomes	• Studies designed to detect and measure nociception-associated biomolecules in human saliva after pain induction AND	• Studies that did not include a pain assessment method
	• compared these to a baseline level before pain induction, either before the onset of acute pain or using a control group	

### Data extraction and data synthesis

Two reviewers independently charted data on article characteristics, study methodology and outcomes. Using a narrative synthesis approach,^9^ included studies are grouped based on the type of biomolecule and the modalities of pain sensation. Variations in outcomes between sexes and correlation between biomolecule concentration change and pain severity are noted where data are available. Papers appear in more than one category if more than one biomolecule was studied.

### Differences between the registered protocol and this review

A more focused analysis was added to examine the evidence for consistent rise in cortisol after cold pain and the pattern of this change. We ran the papers on salivary cortisol and cold pain through an additional set of inclusion criteria where participants were healthy, took no analgesia, and underwent cold exposure shown to be painful as evidenced by increase in pain intensity using a validated tool. Studies were excluded if participants had intentional exposure to another stressor (for example a cognitive task) in the same experiment. If there was more than one arm to the study, only participants not exposed to additional stressors were included.

#### Cortisol-cold pain data synthesis

After contacting study authors, we were unable to obtain data on missing elements (e.g. precise *p*-values, effect size estimates) in a number of studies. This limited the options for data synthesis methodology. To try to determine time of maximum cortisol change after pain induction, we used vote counting based on direction of effect^10^ where outcomes are classified as increase in salivary cortisol (positive direction of effect), decrease (negative direction) or no clear effect (NCE) in 3 defined outcome domains: (a) ≤ 10 min, (b) 10 – 20 min and (c) ≥ 20 min after cold pain induction. In experiments that had multiple time points within a domain, the effect direction was determined using the method described by Hilton, Boon and Thomson (2020). The pre-CPT cortisol concentration at the time point closest to the onset of pain induction was taken as the baseline value. Statistical significance and effect size were not considered in the categorization.^11^

The included studies in the cortisol cold pain analysis were assessed for methodological heterogeneity and risk of bias by two of the authors. Articles were assessed in all 7 domains of Risk of Bias In Non-randomized Studies of Interventions (ROBINS-I).^12^ We added assessment of funding and conflict of interest. In each domain one of three categories (low, moderate, high) of risk of bias judgement was assigned.

## Results

### Selection of sources of evidence

**Figure 1.**
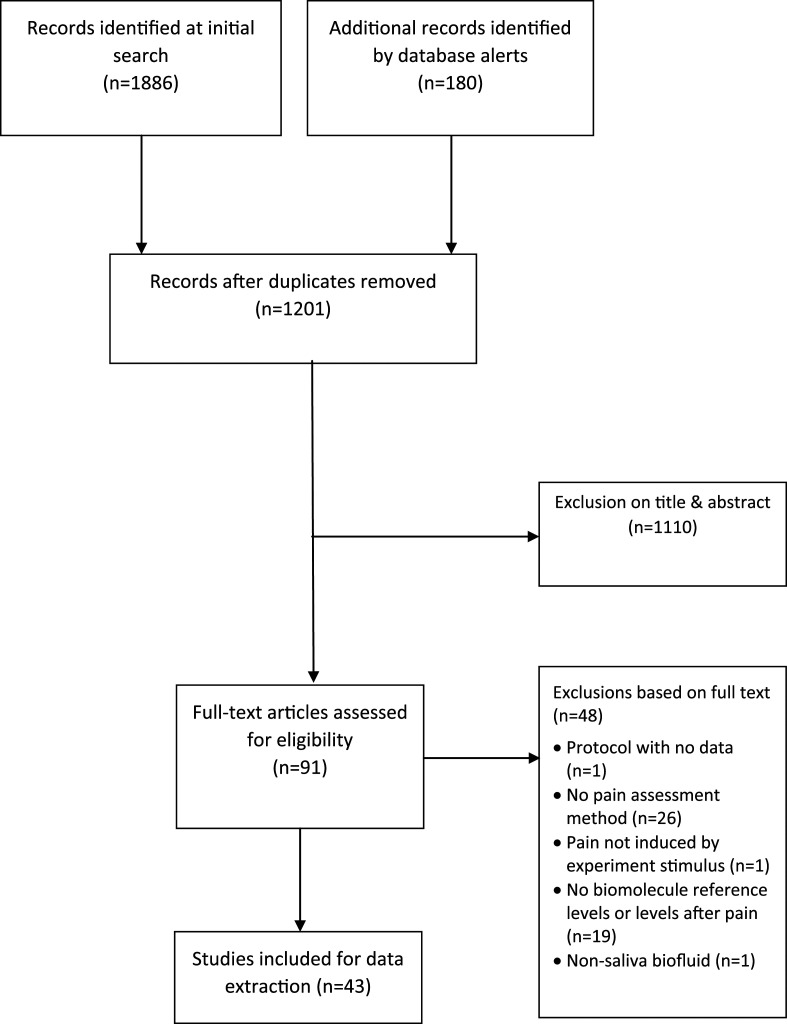
PRISMA flow diagram.

The PRISMA flow diagram appears in Figure 1. The initial search yielded 1886 records and 180 records came through email alerts until 31st December 2022. Ninety-one articles were selected for full text assessment, which were all in English. Forty-three fulfilled the criteria for inclusion (TABLE S1 in supplementary material). No additional articles were identified by searching the reference lists.

### Characteristics of included studies

Most studies (35 of 43) involved experimentally induced pain.^13–46^ The rest involved acute pain after surgery or a medical procedure: hysterectomy,^47^ thoracic surgery,^17^ skin surgery,^48^ corneal surgery,^49^ breast surgery,^50^ gastric or bronchial tube replacement^51^ and drawing blood^52^ or vascular access.^53^ Experimental pain induction was done by cold, heat, ischemia and mechanical, visceral, chemical or electrical stimulation (Table 2). Four studies used more than one pain induction method.^29,30,41,54^ Twenty-eight researchers used standardized methods with citation.^13–16,19–22,24,25,27–32,34–39,42–46,54^ Seven used either a novel technique or a known technique with no citation.^18,23,26,33,40,41,55^ In 41 studies, baseline and post-pain biomolecule concentrations were measured from the same participant group in a before-after design. Two studies used measurements from a control experiment as reference.^40,54^

**Table 2.** Summary of the experimental pain induction methods used.

Type of pain	Pain induction method	Description of method			Articles
Cold pain	Cold pressor task *n* = 18	Immersion of a body region in a cold water bath for a pre-specified maximum time of cold exposure or for as long as the subject could tolerate it			[4] al’ Absi 2003^a, c, g^ [3] al’ Absi 2002^a, c, g^
		Water temperature			[6] Bachmann 2003^a, d, h^ [27] Goodin 2012 (1)^a, c, j^ [31] Hengesch 2018^a, d, h^
		^a^ 0–5			
		^b^ 8–10			
		Body region immersed			[42] Larra-Cinisomo 2015^a, c/d, h^
		^c^ Hand or arm			[54] Nakajima 2011^a, c, g^
		^d^ Foot or feet			[57] Niedbala 2018 ^a, c, h^
		Endpoint (sec)			[66] Serrano 2019^a, c, f^
		^e^45	^f^60	^g^90	[28] Goodin 2012 (2) ^a, c, j^
		^h^180	^i^240	^j^300	[29] Goodin 2012(3) ^a, c, j^
					[62] Quartana (2010) ^a, c, e^
					[79] Youssef (2018) ^a, c, j^
					[1] Archey 2019^a, c, j^
					[12] Burns (2004) ^b, c, i^
					[16] Cruz-Almeida (2004) ^b, d, f^
					[18] Finke (2021) ^a, d, h^
					[47] Lukacs (2022)^a,c,g^
	Plunge test *n* = 1	Intermittent immersion of hand and forearm in a cold water bath of 5°C with duration of immersion and rest periods of 5, 10, or 15 sec			[80] Zimmer 2013
Heat pain	Heat pain threshold *n* = 10	Administration of heat stimuli on the ventral forearm with a thermal device at 35–52°C for 6–50 sec or until pain tolerance was reached			[19] Gaab 2016
					[23] Geva 2014
					[24]Geva 2017
					[25] Geva 2018
					[53] Muhtz 2013
					[62]Quartana 2010
					[76]Wittwer 2016
					[9] Benson 2019
					[26] Geva 2022
					[67] Schneider 2022
	Hot water task *n* = 3	Immersion of hand or arm in a bath of circulating hot water (46–47°C) for 2 min, or for as long as could be tolerated, with a maximum pain exposure of 5 min			[51] Meeus 2008
					[28] Goodin 2012 (2) [29] Goodin 2012 (3)
Mechanical or visceral pain	Mechanical pressure *n* = 6	Application of algometer on at increasing force rate until pain threshold was reached or at 10 N force for 2 min			[21] Geiss 2012
					[34] Hoeger Bement 2010
					[62] Quartana 2010
		Balloon rectal distensions at a pressure of 2–55 mmHg			[9] Benson
					[35] Icenhour 2020
		Lying on the back on a bed of sharp-edged plastic nails (Shakti-mat) for 20 min			[58] Olsson 2011
Ischemic pain	Ischaemic pain task *n* = 2	Modified submaximal effort tourniquet procedure, exercising the hand while blood flow to the arm is occluded for as long as tolerated, with a maximum task duration of 15 min			[28] Goodin 2012 (2) [29] Goodin 2012 (3)
Chemical stimulation	Saline muscle injection *n* = 1	Intra-muscular injection of 0,4 ml hypertonic saline solution into the masseter muscle over about 30 sec			[13] Christidis 2020
Electric stimulation	Electric shock n = 1	Electric shock stimuli using a bipolar cutaneous electrode stimulator on the volar forearm; 20-impulse train of electroshocks with a duration of 5 ms to produce pinching pain			[56] Nelson 2001

Long term analgesia intake interferes with the biomolecules involved in nociceptive pathways.^56^ In 20 articles participants taking regular analgesia were excluded.^15,18–20,22,23,27–30,34,35,37,38,41,43,44,49,50,54^ Four studies included occasional users of analgesics^16,21,31,33^ and one included participants treated with regular analgesia including opioids.^55^ 16 studies did not report on analgesia intake.^13,14,24–26,32,36,39,40,42,45,46,48,51–53^

There is considerable overlap between nociception-related biomolecules and those associated with stress and chronic health conditions. Twenty articles excluded participants with psychiatric disorders^16,20–30,32–34,37,41,43,48,50^ and two did not.^49,55^ The remaining articles did not report on psychiatric conditions.^13–15,17–19,31,35,36,38–40,42,44–47,51,52^ People with chronic pain were excluded in 19 studies^1,3,17,19–22,24–30,35,38,43,47,49,50^ whereas in 20 studies it was not clear if any participants had chronic pain.^14–16,18,31–34,37,39,40,42,44–46,48,51–54^ Four studies enrolled participants with chronic conditions including back pain,^55^ chronic fatigue,^36^ fibromyalgia,^23^ and temporomandibular disorder^41^ as part of the study design.

### Saliva sampling techniques

Whole saliva is a mixture of secretions from salivary glands plus non-salivary components.^57^ Oral mucosal transudate (OMT), collected from the tissues between the cheeks and gums, derives from passive movement of serum components through the oral mucosa into the mouth.^58^

Although salivary biomolecule concentration can be affected by the method of saliva collection and stimulation of flow,^59^ reporting on these in research studies is inconsistent. Three studies provided no information^38,48,53^ and eight did not clearly report whether saliva was stimulated,^16,21,23,31,33,36,48,55^ although this is of little consequence where the biomolecule concentration is independent of salivary flow (e.g. for cortisol). Seven studies included a restriction of 0.5–3 h on tooth brushing and eating to avoid blood contamination from mucosal micro-injuries.^19,20,28–30,32,44^

Food, alcohol, nicotine and caffeine affect salivary flow.^60,61^ In 9 of the studies no restrictions are reported.^13,17,33,38,42,48,50,53,55^ In 11 studies restrictions were variably applied: food was restricted for 30–120 min (mode 60) and caffeine for 0.5–12 h (mode 12). Smoking and alcohol were more variably restricted, sometimes as length of time, and sometimes as dose. One study was specifically designed for investigating pain in smokers^37^ and three gave no information on smoking.^27,35,53^ Alcohol was restricted as the number of units or “drinks” a day in 7 studies^14,15,21,22,31,37,51^ or by asking participants to avoid intake for 0.5–24 h.^16,18–20,28–30,32,34–36,41,43–45,47,52^ 19 studies did not report on alcohol intake.^13,17,23–27,33,38–40,42,46,48–50,53–55^

### Synthesis of results

#### Melatonin

Melatonin has anti-inflammatory properties and reduces hyperalgesia in animal models. Surgical and cancer patients report higher pain intensity and use more analgesia in the day time though melatonin’s role in these phenomena is unproven.^62^ In a study of healthy people salivary melatonin decreased within 5 min of painful electric stimulation followed by a rise.^38^ Correlation with pain ratings or sex were not analyzed.

#### Kallikreins

Kallikreins are responsible for physiological functions including blood pressure regulation and inflammation.^63^ Increase in salivary kallikrein was shown 2-6 h after hysterectomy. The peak increase was at 4 h but pain ratings did not follow this pattern, peaking 1 hour post-operatively before decline.^47^ No analysis by participants’ sex was done.

#### Secretory immunoglobulin A (sIgA)

There is a relationship between stress, including that induced by CPT, and change in sIgA. There are no clear mechanisms to explain sIgA change in relation to pain.^64^ In a study where pain intensity during CPT was measured, sIgA fell significantly after first exposure to CPT, but not after second exposure in the same participants’ other arm. There was no correlation with pain intensity.^18^

Salivary sIgA was measured in thoracic surgery with or without regional anaesthesia. While there was no difference in pain intensity between the two groups, reduction in sIgA, only occurred in the regional anaesthesia group at the 6 h time point.^17^ Conversely, in patients who underwent corneal surgery, sIgA increased 1 h post-surgery and this rise correlated with pain intensity.^49^ Sex differences were not analysed in either study.

#### Testosterone

Animal studies show that testosterone may have a protective effect in the development of chronic pain.^65^ No change in salivary testosterone was found after corneal surgery^49^ but after thoracic surgery, testosterone increased regardless of whether regional anaesthesia was used for pain control. In a study on female pain perception, no difference in salivary testosterone was found between healthy males and females after CPT.^13^

### Pro-inflammatory cytokines and soluble tumour necrosis factor α receptor II (sTNF-αRII)

Pro-inflammatory cytokines have a role in the development of neuropathic pain.^66^ In a study that measured change in four cytokines (IL-6, IL-8, IL-10, IL-4) in the saliva and blood of healthy participants, after CPT or a painless thermal task, cytokine concentrations peaked 45-60 min after CPT while no change occurred in the control experiments.^20^ The time course of cytokine change was nearly identical in saliva and plasma. In another study, pressure pain thresholds were measured in defined anatomical points in women with fibromyalgia and pain-free women. Salivary IL-6 (and cortisol) increased after pain pressure in patients with fibromyalgia but not in healthy subjects.^23^

TNFα receptor 2 (TNFαR-II) has a neuroprotective role. Soluable TNFαR-II is the circulating form of this membrane bound receptor. In all three studies that analyzed salivary sTNFαR-II, there was reduction in the levels after acute pain.^29,30,49^ Two were studies in healthy volunteers after exposure to multiple pain modalities (cold, heat and ischaemic pain). sTNFαR-II fell either immediately after pain induction or 25-35 min later.^29,30^ sTNFαR-II also fell 1 hour after corneal surgery.^49^ There was no significant correlation with pain ratings in the 2 studies that analysed this.^29,30^ None of the studies reported analysis by participants’ sex.

### Salivary alpha-amylase

Salivary alpha-amylase (sAA) increases in response to sympathetic over-activity.^67^ We found 13 acute pain studies that assayed sAA.^17,19,22,34,42–45,49–51,53,54^ No change was found in healthy participants after painful hypertonic saline muscle injection.^19^ One heat pain experiment reported rise in sAA correlating with pain intensity^44^ but in two studies designed to observe the impact of psychosocial stress on pain perception, heat pain alone was not associated with change in sAA, while psychosocial stress was.^22,43^ Similarly, in a study that examined the effect of hydrocortisone vs placebo on heat and visceral pain, there was no rise in sAA (or cortisol) after pain induction in the control arm.^54^

Rise in sAA after cold pain in healthy participants was showed in 2 studies but correlation with pain intensity was not analysed.^34,45^ Change in sAA after CPT is affected by catechol-O-methyltransferase (COMT) Val158Met polymorphism where greater change has been found in Met allele carriers though pain ratings were equal in the groups.^42^ sAA rise also occurred in people with severe disabilities undergoing medical procedures, correlating with pain intensity,^51^ and after thoracic surgery^17^ but no rise was found after painful vascular access,^53^ corneal or breast surgery.^49,50^

### Cortisol

Cortisol is the most studied salivary biomolecule in relation to nociception.^14–17,21–37,39–43,46,48,49,52,54,55^ Thirty two studies in this review have measured salivary cortisol and in most (*n* = 26), pain was experimentally induced.^14–16,21,22,24–32,34,35,37,39–41,43,46,48,49,54,55^ In the induced studies, six found no difference between men and women^14,27,28,31,32,34^ and one reported a greater cortisol rise in men.^46^ In 3 studies a positive correlation was found between cortisol change and pain intensity ratings,^28,29,46^ while five studies found no such correlation.^22,36,40,41,43,55^ The rest did not report any analyses with respect to sex or pain intensity.

#### Post-operative and post-procedure pain

Salivary cortisol levels were at the high end of the normal range immediately before drawing blood, thereafter declining (after venipuncture) or staying the same (after finger prick).^52^ There was rise in salivary cortisol 30 min after skin surgery compared with 1 week before the operation, but not when compared to 30 min pre-operatively.^48^ Cortisol increased in the immediate pre-operative period compared to baseline in people having corneal surgery, with a further rise 1 h post-surgery.^49^

After thoracic surgery, cortisol increased compared to a baseline taken at the time of qualification for surgery, regardless of the provision of regional anaesthesia.^17^ Importantly, salivary cortisol was not measured in the immediate pre-operative period in this study. None of the surgical studies analyzed correlation with sex. Correlation with pain intensity after drawing blood and thoracic surgery were analyzed and were not significant.

#### Heat pain

Salivary cortisol (and sTNFαR-II) were measured in two studies, where healthy volunteers were exposed to multiple pain modalities including cold, heat and ischaemic pain tasks.^29,30^ In one of these, salivary cortisol elevation occurred after a battery of painful tasks.^30^ In the other, biomolecule changes were analysed separately, with the finding that heat pain (and ischaemic pain) alone did not induce change in salivary cortisol while CPT did.^29^ Similarly, in six studies designed to assess the effect of acute psychosocial stress on pain modulation, heat pain alone or in combination with a sham stress task, was not associated with change in cortisol.^22,24–27,43^ It was psychosocial stress that predicted cortisol rise. Correlations with sex were not analyzed except in one study where the researchers found that women exhibited stress-induced anti-nociception and men exhibited stress-induced pro-nociception.^27^ Correlation with pain intensity was not analysed in these experiments.

In comparisons of healthy subjects with participants who had chronic pain, fatigue or depression, no increase was found in salivary cortisol after heat pain induction in any of the groups.^36,55^

#### Mechanical & visceral pain

No change was observed in salivary cortisol in healthy participants lying on a bed of nails compared to lying on a soft bed, despite participants lying on nails reporting rapid rise in pain.^40^ Similarly, salivary cortisol did not change from baseline after applying painful pressure to the index finger of healthy participants but there was rise in cortisol if they were due to do a cognitive ‘stressor’ mathematics task.^32^

In women with fibromyalgia salivary cortisol (and IL-6) increased after measuring pain pressure thresholds but this did not happen in pain-free women.^23^ In a comparison of people with temporomandibular disorder with healthy controls, pain pressure thresholds were measured, along with heat and cold pain thresholds. There was no difference in cortisol response between the two groups.^41^

In a study of visceral pain induced by rectal distension in healthy individuals, the results of salivary cortisol change were analyzed according to whether participants had high or low perceived background stress. Cortisol levels were higher throughout the experiment in those with higher perceived stress but there was no rise in cortisol in either group.^33^ Similarly, there was no rise in salivary cortisol (or sAA) after visceral and heat induction the placebo arm of a trial examining the effect of hydrocortisone vs placebo on pain perception.^54^

#### Cold pain

In studies that measured salivary cortisol, cold pain was induced using CPT^14–16,21,28–31,34,35,37,39,41,42^ or the plunge test.^46^ Four of the cortisol-cold pain studies were excluded from the direction of effect analysis because either pain was induced by a combination of stimuli with no separate analysis of cold pain,^30,41^ or, the experimental design included an emotional or cognitive task not separated from cold induction.^31,39^ In one of these, where participants were put in a situation that allowed positive appraisal of cold pain, the cortisol response was inhibited compared with controls, though pain intensity was the same.^39^ The other study showed that the cortisol response to CPT combined with a cognitive task was blunted in people with early life adversity though they experienced the same pain intensity as controls.^31^ In a study where half of the healthy participants were exposed to social stress and the others were not, salivary cortisol increased in both groups after CPT.^15^ Participants exposed to social stress reported less pain but had greater cortisol rise. The 76 participants of this study who were not exposed to social stress met the inclusion criteria for the direction of effect analysis. Overall these results suggest a disconnection between the salivary cortisol response to cold and pain intensity.

11 articles met the inclusion criteria for a more focused review of the effect of cortisol on cold pain.^14–16,21,28,29,34,35,37,42,46^ In one of these, hand CPT and foot CPT were done separately in the same participants.^34^ This study was therefore entered as 2 experiments, giving 12 experimental study groups for the analysis. Therefore a total of 576 individual cold pain experiments (mean participant age 23.2 years) were included in total, with 11 drop outs. The effect direction plot is presented in Table 3. Measurements after 20 min were only taken in six experiments. Ten experiments had data in the less than 10-min outcome domain and ten in the 10–20 min outcome domain. Increase in salivary cortisol is reported in most experiments 10–20 min after cold pain induction.

**Table 3.** Effect direction plot summarizing direction of change in salivary cortisol levels from studies of experimental cold pain induction.

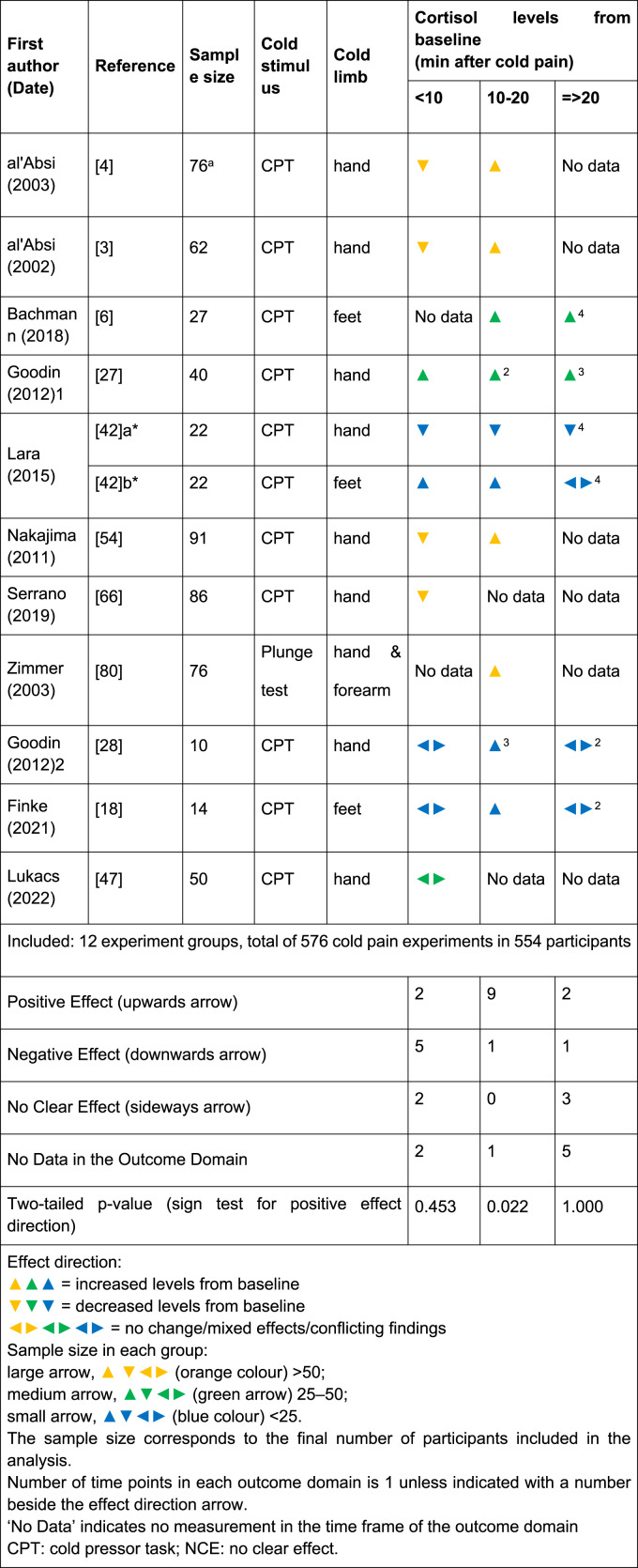

### Risk of bias (ROB)

A ROB table is presented (Table 4). The important confounder would be co-exposure to psychological stress which would falsely create, or amplify rise in salivary cortisol. All experiments with no control (neutral or warm water) were judged at least moderate in risk of confounding. A reasonable step to minimise stress would be participant awareness that they could withdraw at any time. If no steps were taken to minimise stress the risk was judged high. Where there was a control, risk was judged to be low but only if stress and anxiety were showed to be equal in cold water and control groups, and, did not rise in the control group after the task. If this was not shown, the risk was judged moderate.

**Table 4.** Risk of bias in included studies.

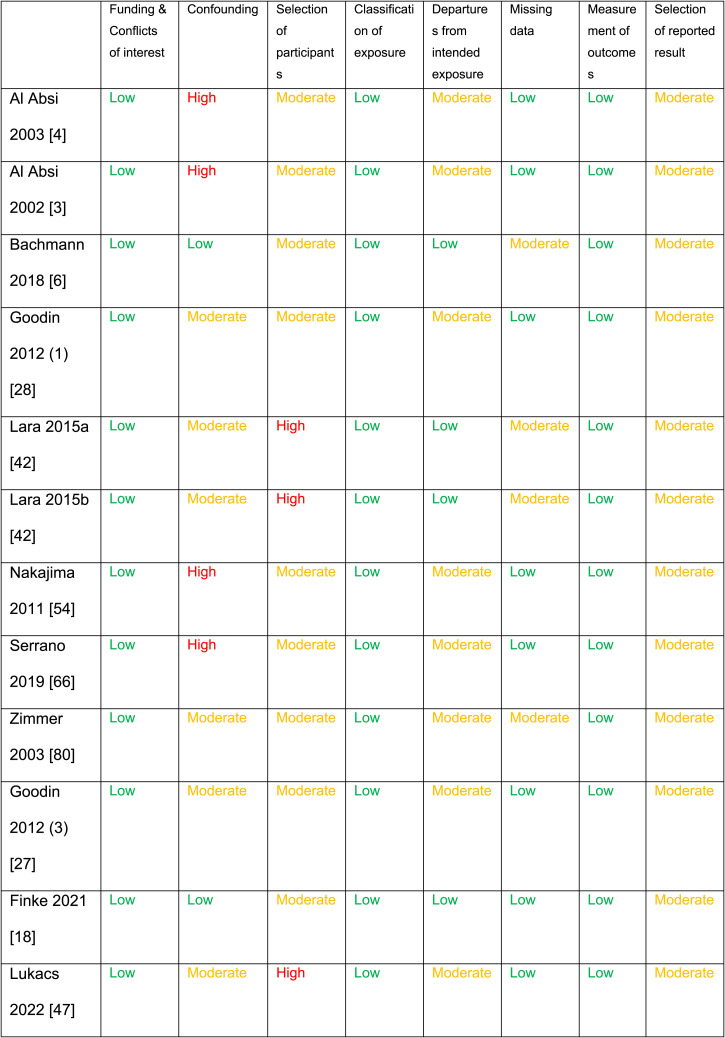

Selection bias is principally related to recruitment being restricted to university communities. This was judged to be at least moderate in all the experiments. It was judged high when it was unclear whether all potential participants had an equal chance of inclusion. This bias creates issues of generalizability or transferability to other populations, and could be classified as sampling (rather than selection) bias. Nonetheless, we included it because of a concern that it is ignored by many: only 4 of the 11 papers mentioned this bias in their discussion section.

ROB related to classification of exposure was judged low in all experiments because the exposures to cold and control procedures were well defined prior to the outcome assessment.

We considered experimenter and participant interaction to be a co-exposure that could affect change in cortisol concentration. ROB due to departures from intended exposure was judged moderate when these interactions were not clearly standardised for example it was unclear whether the experimenter was in the room during CPT.

ROB due to missing data was judged low when there were no missing data in relation to salivary cortisol measurement or researchers accounted for this in the analysis. Where this was not accounted for, ROB was considered moderate because there was no indication of differential loss related to prognostic factors. Overall 576 individual experiments were done, where 19 (3.3%) had missing data relevant to pain induction and salivary cortisol measurement, with 11 (1.9%) not accounted for in study analyses.

Samples were stored at −20, −70 or −80°C before defrosting in bulk for analysis sometime later. Although none of the papers described blinding at the analysis stage, we considered performance bias unlikely and ROB in measurement of outcomes was judged low.

We were not able to access pre-specified protocols for any of the included studies. All researchers used only one method of cortisol measurement and results analysis and values were not selected from multiple outcomes. ROB in selection of the reported result was judged moderate for all the studies.

All studies were funded by non-profit organisations, university funding bodies or national institutes and were at low risk of funding bias. Two papers specified the role of the funders in the conduct of the research and its publication, both reporting no role.^29,34^

Overall the risk of bias was judged high in six studies (7 experiments) and moderate in 4. High risk was due to possible confounding in 4 studies and due to possible selection bias in two (3 experiments).

### Methodological heterogeneity in salivary cortisol cold pain studies

#### Differences in saliva collection

##### Timing of collection

In all but one article, experiments^35^ were conducted in a particular part of the day: three were done in the morning^14,15,37^ and 7 in the afternoon.^16,21,28,29,34,42,46^ In 4 articles, no reason was given for this choice,^14,15,34,37^ 2 stated that afternoon times are associated with greater cortisol responses^28,29^ and others simply stated ‘to control for diurnal variation’.^16,21,42,46^

##### Collection method

Whole saliva was collected in 11 experiments and oral mucosal transudate in one. The device used was usually a cotton swab that was later centrifuged to release saliva.^14–16,21,28,29,35,37,46^ This method can yield a different cortisol concentration compared to saliva obtained by passive drool.^68^ As salivary cortisol closely follows free serum cortisol, this is unlikely to be significant for this data synthesis.

##### Participant preparation

Restrictions to food, alcohol, smoking and caffeine were variably applied. Most researchers placed restrictions on all of these.^14–16,21,28,29,34^ One study placed no restrictions,^42^ one restricted alcohol only^37^ and one restricted smoking only.^46^ Precautions to reduce the risk of blood contamination from gums were taken in four experiments.^16,28,29,34^

#### Differences in assays of salivary cortisol concentration

Immuno-assays with high sensitivity were used. Intra and inter-assay coefficients of variation were reported in six of the included articles with values ranging 4%–12%.^16,28,29,34,37,46^

#### Differences in conducting cold pain induction tests

Overall the experiments had little heterogeneity with respect to the conduct of CPT. Water temperature was 0°C–5°C. Nine experiments were of upper limb immersion and 3 were feet immersions.

## Discussion

Many salivary biomolecules have been studied in acute pain settings. Researchers’ rationale for selecting these biomolecules varies. Melatonin, cytokines and testosterone were selected because of evidence for their involvement in modulation of noxious stimuli. Kallikriens, sAA and sIgA have been selected because they are stress biomarkers. Cortisol is a stress marker and is released in response to acute pain through HPA axis activation.^69^ Some of these salivary biomolecules have been studied in very few experiments and the most commonly studied are sAA and cortisol. Regardless of the type of biomolecule, there is considerable methodological variation in the studies. Most researchers have chosen to induce pain under controlled conditions with cold pain induced by CPT being the most studied modality.

Salivary biomolecules that change with stress would be expected to change after acute pain in healthy individuals. This expectation is not consistently met, but when pain modalities have been compared in salivary cortisol experiments, change is encountered after controlled pain induction with cold rather than other modalities including heat, ischaemic, pressure or visceral pain.

In experiments designed to differentiate between responses to pain and stress, stress is found to be a better predictor of sAA and cortisol rise. This may explain the inconsistent relationship between the magnitude of cortisol or sAA rise and pain severity. Exposure to stressful cognitive or psychosocial tasks combined with heat or pressure pain, results in rise in sAA and cortisol while heat or pressure pain alone do not. In contrast, in a study involving healthy people, positive appraisal of cold pain reduced the stress response, including a lack of rise in salivary cortisol.

In the same vein, where salivary cortisol has been measured after surgery or procedures, regardless of the great variation in the physical nature of the painful interventions, its rise is timed more to pre-operative or pre-procedure stress than the ensuing trauma and pain.

There are complex relationships between gonadal hormones and pain processing.^70^ and in women, menstrual cycle phase and pregnancy can influence cortisol concentration. Some researchers have circumvented these effects by recruiting only male participants.

Of 43 articles included, 17 analyzed the relationship between biomolecule concentrations and pain intensity and only 11 analyzed the relationship with sex. The groups are highly heterogeneous and it is not possible to draw reliable conclusions from them.

Looking more closely at 12 experiments where salivary cortisol was measured after experimentally induced cold pain in healthy people, it is possible to cautiously suggest that salivary cortisol rises 10-20 min after cold pain induction. This caution is advised because more than half of the experiments are judged to be at high risk of bias (though only one domain carries this high risk in each of these) and most papers had missing elements such as precise *p* values and effect size estimates which precluded reliable quantitative analysis.

Heterogeneities in methodology influence the magnitude of change in cortisol. These include differences in the timing and method of saliva collection, blood contamination, restrictions on substances that blunt or enhance the cortisol response, exercise, the assay used, and conduct of cold pain induction. Alcohol, nicotine and caffeine are commonly used substances that affect salivary flow.^60,61^ As salivary cortisol is not affected by flow, this aspect would not influence the results of the cortisol–cold pain data synthesis. The effects of these substances, and also food and exercise, on cortisol secretion are potentially more important. The effect of exercise varies depending on whether it is regular or done in acute bouts.^71^ Caffeine and nicotine are HPA stimulators^72–74^ though the cortisol response is blunted in habitual smokers.^75^ Alcohol consumption is associated with higher daily circulating cortisol levels but the stress response is suppressed with habitual high intake.^76^ Therefore these substances either blunt or enhance the cortisol response, influencing how easily it would be detected.

The effect of the circadian rhythm on the cortisol response to pain is not known. Regardless of the timing, most researchers did not explain the reason for their time choice clearly and may have been influenced by convenience factors such as participants’ availability or laboratory space. Cortisol is not the only nociception related biomolecule with a circadian rhythm. An obvious other example is melatonin and there may be other, hitherto unrecognized, patterns of diurnal change.

There is considerable methodological variation in inducing pain experimentally under controlled conditions with unknown consequences on the magnitude of biomolecule changes. We found this to be the case in all modalities, even cold pain induced by CPT, where we expected a relatively standardized approach. Variations have developed to the original CPT design,^77^ including immersion of the non-dominant hand, hand plus forearm, one or both feet^16,34^ or single finger.^78^ They all induce a physiological response with some evidence for a relationship between the response magnitude and the surface area of cooled skin.^34,78^ Additionally differences have been found in sympathetic responses to lateralized cold stimuli.^79^ Although the cortisol response has not been studied in this way, some researchers argue for bilateral feet cold stimulation to avoid laterality bias and to keep arms free for other purposes (e.g. blood sampling).^16,31,34^ Additionally, we have found other variations in the conduct of cold pain induction for example the exact water temperature and test end points.

Participants’ mean age in the included studies is relatively young. Daily cortisol output increases with age^80^ but the effect on the cortisol stress response is unknown. There is less knowledge on age related effects for other biomolecules, an important gap in the literature.

A highly heterogeneous and complex landscape has developed in this research field. To be useful in clinical practice as a guide to acute pain treatment, the ideal salivary nociception biomarker would be one (or a panel of biomarkers) that changes reliably after noxious stimuli, within a short time interval of at most a few minutes, in healthy people and in those with acute or chronic conditions. It should be either minimally or predictably affected by change in the organism’s internal or external environment. There remain considerable challenges in identifying such biomarkers. Importantly, there are differences in salivary biomolecule responses to different pain modalities and none of the biomolecules studied to date are specific to nociceptive pathways.

Improvements in bioengineering will enable measurement of salivary biomolecules more easily and at lower cost. To advance this area of research, it is essential to standardize methodology in salivary sample collection and pain induction. Salivary biomolecule secretion is affected by a complex multitude of factors in both healthy individuals and those with physical and mental health disorders or chronic stress.

Researchers should be aware of the wider factors that can affect biomolecule concentration such as salivary flow, commonly used pharmacological substances, exercise, acute stress, chronic conditions including chronic pain and psychiatric conditions, use of analgesia, diurnal variations and participant demographics. Cortisol secretion in particular is influenced by many of these. There can therefore be large differences in biomolecule levels that are not merely due to measurement error or individual variation. This difficulty can be augmented by the lack of consistency between different assays. In experimental designs, measuring change in biomolecule levels is likely to be more informative than absolute levels. Less heterogeneous experimental designs should be agreed and implemented by the researchers in this field in order to create a more cohesive and clinically relevant research literature.

## Supplemental Material

Supplemental Material - Nociception related biomolecules in the adult human saliva: A scoping review with additional quantitative focus on cortisol Supplemental Material for Nociception related biomolecules in the adult human saliva: A scoping review with additional quantitative focus on cortisol by Roxaneh Zarnegar, Angeliki Vounta, Qiuyuan Li and Sara S Ghoreishizadeh in Molecular Pain.
