# Supplementary material for: Nociception related biomolecules in the adult human saliva: A scoping review with additional quantitative focus on cortisol
Source: Mol Pain. 2024 Mar 5;20:17448069241237121. doi: 10.1177/17448069241237121 (PMC10916496; doi:10.1177/17448069241237121)
Supplement: Supplemental Material - Nociception related biomolecules in the adult human saliva: A scoping review with additional quantitative focus on cortisol [file sj-pdf-1-mpx-10.1177_17448069241237121.pdf]

TABLE S1: Characteristics of included studies (n = 43).
Risk of bias summary for articles included in the cortisol – cold pain quantitative synthesis
Abbreviations: Indirect antibody enzyme-linked immunosorbent assay (ELISA). Where mean age was not reported, any other information about age was inserted in the table. Only significant outcomes are inserted in the table.

Ref	First Author (publication year)<br>Biomolecule(s)	Characteristics
15	Alabsi (2003)<br>Cortisol	<u>**AIM**</u><br>To determine the extent to which hemodynamic and cortisol changes during acute psychological stress predict pain perception<br><br><u>**METHODS**</u><br>**Country:** United States of America<br><br><u>**Participants**</u><br>**Recruitment:** From the university community by posters and newspaper advertisements<br>**Age:** Mean 20.3<br>**Sex:** 80 F, 72 M<br>**Total number of participants:** 152<br>**Dropouts:** 0<br>**Reason for drop out:** Not applicable<br>**Revised sample size:** 152<br>**Analgesia intake:** None<br>**Chronic conditions:** Healthy (psychiatric conditions and chronic pain not specifically reported)<br>**Restrictions:** Food, alcohol, smoking, caffeine<br><br><u>**Study design:**</u><br>Salivary cortisol measured before and after an induced noxious cold stimulus applied after a psychosocial stress (public speaking) or rest condition<br>**Other measurements:** Pain intensity and quality, mood, blood pressure, stroke volume<br>**Interventions:** Acute cold pain induced using CPT<br>**Comparison:** Change from baseline compared between stress and rest states.<br>**Salivary assay:** Time resolved immunoassay with fluorometric end point detection<br>**Saliva type:** Unstimulated saliva<br>**Collection method:** Swab<br><br><u>**OUTCOMES**</u>

		Salivary cortisol increased in both groups after CPT. Participants in the social stress arm of the experiment reported less pain but salivary cortisol rise was greater<br>**Correlation with pain ratings:** Not analysed<br>**Sex effects:** Not analysed<br><br>**<u>NOTES</u>**<br>**Inclusion in the cortisol- cold pain quantitative analysis:** Yes, for the 76 participants not exposed to the stress task<br><br>**<u>BIAS</u>**<br>**Bias Type:** High risk of confounding. Moderate risk in selection of participants, departures from intended exposure and selection of reported results<br>**Author's judgement:** High ROB<br>**Support for judgement:** No control arm and no reported measures to reduce participant anxiety, recruitment limited to university community, interactions between experimenter and participant not clearly defined and no published pre-specified protocol
14	Alabsi (2002) Cortisol	**<u>AIM</u>**<br>To evaluate the extent to which cortisol concentrations, blood pressure and hemodynamic contribute to gender differences in pain sensitivity has not been investigated<br><br>**<u>METHODS</u>**<br>**Country:** United States of America<br><br>**<u>Participants</u>**<br>**Recruitment:** From the university community by posters and newspaper advertisements<br>**Age:** 19.7<br>**Sex:** 34 F, 31 M<br>**Total number of participants:** 65<br>**Dropouts:** 3 (2F, 1 M)<br>**Reason for drop out:** Not applicable<br>**Revised sample size:** 65<br>**Analgesia intake:** Not reported<br>**Chronic conditions:** Healthy (psychiatric conditions and chronic pain not specifically recorded)<br>**Restrictions:** Food, alcohol, smoking, caffeine<br><br>**<u>Study design:</u>**<br>Salivary cortisol measured before and after an induced noxious stimulus<br>**Other measurements:** Blood pressure, heart rate, stroke volume, pain intensity, pain descriptors (MPQ), mood<br>**Intervention:** Acute cold pain induced using CPT

		**Comparison:** Change from baseline<br>**Salivary assay:** Time-resolved immunoassay with fluorometric end point detection<br>**Saliva type:** Unstimulated<br>**Collection method:** Swab<br><br>**<u>OUTCOMES</u>**<br>Salivary cortisol increased following the CPT<br>**Correlation with pain ratings:** Not analysed<br>**Sex effects:** No correlation found<br><br>**<u>NOTES</u>**<br>Women reported greater pain than men during and after CPT.<br>Cortisol concentrations predicted lower pain reports during and after CPT in men only.<br>**Inclusion in the cortisol- cold pain quantitative analysis:** Yes<br><br>**<u>BIAS</u>**<br>**Bias Type:** High risk of confounding. Moderate risk in selection of participants, departures from intended exposure and selection of reported results<br>**Author's judgement:** High ROB<br>**Support for judgement:** No control arm and no reported measures to reduce participant anxiety, recruitment limited to university community, interactions between experimenter and participant not clearly defined and no published pre-specified protocol
13	Archey (2019) Testosterone	**<u>AIM</u>**<br>To examine the role of testosterone in female cold pain expression and perception<br><br>**<u>METHOD</u>**<br>**Country:** United States of America<br><br>**<u>Participant</u>**<br>**Recruitment:** Recruited from an undergraduate university using flyers and campus-distributed<br>**Age:** Mean 21.61<br>**Sex:** 38 F, 16 M, 2 not self-identified on questionnaire<br>**Total number of participants:** 56<br>**Dropouts:** 10 (8 medication use or medical conditions, 2 incomplete sex identification on question sheet)<br>**Reasons for drop out:** Medication use, medical conditions known to affect hormone levels<br>**Revised sample size:** 46 (32 F, 14 M)<br>**Analgesia intake:** Not reported<br>**Chronic conditions:** Healthy (psychiatric conditions not specifically reported, chronic pain part of exclusion criteria)

		**Restrictions:** None reported<br><br>**<u>Study design:</u>**<br>Salivary testosterone measured before and after an induced noxious stimulus<br>**Other measurements:** Pain intensity<br>**Intervention:** Cold pain induced by CPT<br>**Comparison:** Change from baseline<br>**Salivary assay:** ELISA<br>**Saliva type:** Stimulated<br>**Collection method:** Passive drool<br><br>**<u>OUTCOMES</u>**<br>No significant difference in salivary testosterone between males and females<br>**Correlation with pain ratings:** No correlation found<br>**Sex effects:** No significant difference between males and females
48	Augustin (1999) Cortisol	**<u>AIM</u>**<br>To investigate the peri-operative stress reactions following skin surgery by assessing cortisol, anxiety, vital functions and immune parameters<br><br>**<u>METHOD</u>**<br>**Country:** Germany<br><br>**<u>Participants</u>**<br>**Recruitment:** Consecutive patients with nevi from a dermatology clinic<br>**Age:** Mean 33.9<br>**Sex:** 25 F, 20 M<br>**Total number of participants:** 58<br>**Dropouts:** 13<br>**Reason for drop out:** Not fully reported (most frequent reason stated to be “fear of blood-drawing”)<br>**Revised sample size:** 45<br>**Analgesia intake:** Not reported<br>**Chronic conditions:** Healthy (psychiatric conditions and chronic pain not specifically reported)<br>**Restrictions:** None reported<br><br>**<u>Study design:</u>**<br>Salivary cortisol measured before noxious surgical stimulus, intraoperatively and after surgery<br>**Other measurements:** Blood pressure, heart rate, respiratory rate, lymphocyte subpopulations, pain intensity<br>**Intervention:** Skin surgery under local anaesthesia<br>**Comparison:** Change from baseline (pre-operative) samples<br>**Salivary assay:** Not reported

		**Saliva type:** Unclear<br>**Salivary Collection Method:** Not reported<br><br>**OUTCOMES**<br>Significant rise in salivary cortisol 30 minutes post-surgery compared with 1 week before the operation, but not when compared to 30 minutes pre-operatively<br>No significant difference between 30 minute preoperative and intra-operative levels<br>Cortisol levels remained elevated one week after surgery<br>**Correlation with pain ratings:** Not analysed<br>**Sex effects:** Not analysed<br><br>**NOTES**<br>The reported pain intensity after the surgery was low-moderate in this study. On this basis the authors concluded that the cortisol rise is likely to be related to anxiety rather than pain or pain-induced stress
16	Bachmann (2018) Cortisol	**AIM**<br>To examine the validity and feasibility of a fully automated bilateral feet CPT<br><br>**METHOD**<br>**Country:** Germany<br><br>**Participants**<br>**Recruitment:** Internet announcement posted at the university<br>**Age:** 26<br>**Sex:** Male only<br>**Total number of participants:** 28<br>**Dropouts:** 1<br>**Reason for drop out:** Missing samples for cortisol analysis (1), problems with haemodynamic data (2)<br>**Revised sample size:** 27 for cortisol analysis, 26 for haemodynamic analysis<br>**Analgesia intake:** Yes<br>**Chronic conditions:** Healthy (psychiatric conditions part of exclusion criteria, chronic pain not specifically reported)<br>**Restrictions:** Food, alcohol, smoking, caffeine. Precautions to reduce risk of contamination due to bleeding from gums<br><br>**Study design:**<br>Measurement of salivary cortisol before and after exposure to noxious cold stimulus or warm water control<br>**Other measurements:** Pain intensity, stress, arousal, anxiety, haemodynamic data (blood pressure, heart rate, stroke volume, left ventricular ejection fraction, cardiac output, total peripheral resistance,

		**Comparison**: Change from baseline compared between CPT and control groups<br>**Intervention:** Acute cold pain using CPT or warm water control<br>**Salivary assay:** Time-resolved immunoassay with fluorometric detection<br>**Saliva type:** Unclear<br>**Saliva collection method:** Swab<br><br>**<u>OUTCOMES</u>**<br>**Correlation with pain ratings:** Not analysed<br>**Sex effects:** Not analysed<br><br>**<u>NOTES</u>**<br>**Inclusion in the cortisol- cold pain quantitative analysis:** Yes<br><br>**<u>BIAS</u>**<br>**Bias Type:** Moderate risk in selection of participants, missing data missing data and selection of reported results<br>**Author's judgement:** Moderate ROB<br>**Support for judgement:** Recruitment only from the university community, missing data excluded in the analysis (but no indication that there was differential loss of data) and no published pre-specified protocol
54	Benson (2019) Salivary alpha amylase	**<u>AIM</u>**<br>To test the effects of oral hydrocortisone on pain thresholds and explore the sex differences as well as the effects of hydrocortisone on pain related fear<br><br>**<u>METHOD</u>**<br>**Country:** Germany<br><br>**<u>Participants</u>**<br>**Recruitment:** By local advertisement<br>**Age:** hydrocortisone arm: mean 24.8, placebo arm: mean 25.1<br>**Sex:** 50 F, 50 M<br>**Total number of participants:** 108<br>**Dropouts:** 8<br>**Reasons for drop out:** medical condition or medication intake (5), technical issues on the study (3)<br>For rectal distension: distention pressure limit precluded measurement of the threshold (8 in hydrocortisone group, 7 in placebo group and in the heat pain 4 excluded (no reliably determined heat thresholds 3 shared with the other 15.<br>**Revised sample size:** 100<br>**Analgesia intake:** None (irregular use of over the counter pain medications permitted)<br>**Chronic conditions:** Healthy (psychiatric conditions and chronic pain not reported)

		**Restrictions:** smoking<br><br><u>**Study design:**</u><br>Measurement of salivary cortisol and alpha-amylase before and after induced noxious visceral and heat stimuli with pain induction taking place before and after hydrocortisone or a placebo control pill, in double blind randomized trial<br>**Other measurements:** pain intensity<br>**Interventions:** Combined pressure-controlled rectal distension (barostat system) & heat pain<br>**Comparison:** Comparison of biomolecule levels at different experimental time points between hydrocortisone and placebo arms<br>**Salivary assay:** Cortisol: ELISA. Alpha amylase: Saliva Enzymatic Assay<br>**Saliva type:** Not recorded<br>**Salivary Collection method:** swab<br><br><u>**OUTCOMES**</u><br>No rise in amylase or cortisol after pain stimuli in the placebo arm of the trial<br>**Correlation with pain ratings:** Not analysed in the control arm<br>**Sex effects:** Not analysed in the control arm<br><br><u>**NOTES**</u><br>Heat pain thresholds were not affected by hydrocortisone<br>Hydrocortisone decreased the pain threshold for visceral pain and this was primarily driven by women
17	Bialka 2021<br>Cortisol<br>Testosterone<br>sIgA<br>Alpha-amylase	<u>**AIM**</u><br>To assess the effectiveness of thoracic paravertebral regional block for post-operative pain after video-assisted thoracic surgery (VATS) compared with no block<br><br><u>**METHOD**</u><br>**Country:** Poland<br><br><u>**Participants**</u><br>**Recruitment:** Details not reported<br>**Age:** Mean age of paravertebral block group: 64, control: 61<br>**Sex:** paravertebral block group: F 21, M 16. Control: F 16, M 17<br>**Total number of participants:** 119<br>**Dropouts:** 49<br>**Reason for drop out:** 7 did not meet inclusion criteria, 2 declined participation, 19 conversion to open procedure, 7 re-operation, 6 ineffective block, 8 data lost<br>**Revised sample size:** 70 (study group: 37, control: 33)<br>**Analgesia intake:** post-operative patient-controlled analgesia with oxycodone, no preoperative analgesia

		**Chronic conditions:** Participants could have a range of health conditions within American Society of Anesthesiology physical status I-III (psychiatric conditions not specifically excluded, chronic pain part of exclusion criteria)<br>**Restrictions:** None reported<br><br>**<u>Study design:</u>**<br>Salivary cortisol, testosterone, sIgA & alpha-amylase measured before and after surgery in a randomised study comparing thoracic paravertebral block and a control group with no block<br>**Other measurement:** Pain intensity, blood pressure, heart rate<br>**Interventions:** VATS<br>**Comparison:** Change from baseline compared between study (regional block) and control groups<br>**Salivary assay:** Cortisol & testosterone : Commercial ELISA. sIgA: Commercial ELISA kits. Alpha-amylase: static method with an AMYLAZA kit<br>**Saliva type**: Mixed stimulated and unstimulated<br>**Salivary Collection method:** Swab<br><br>**<u>OUTCOMES</u>**<br>There was increase in all the biomolecules measured in the study 6 hours and also 24 hours after surgery compared with pre-operatively with the exception of sIgA which fell in the regional block group between at the 6 hour time point. There was no significant difference in this change between the intervention and control groups.<br>**Correlation with pain ratings:** Alpha-amylase levels were significantly associated with higher one month pain intensity score<br>**Sex effects:** Not analysed
18	Burns (2004)<br>sIgA	**<u>AIM</u>**<br>To investigate whether differences in the timing of saliva are the explanation for discrepant results in change in salivary IgA after acute stress tasks<br><br>**<u>METHOD</u>**<br>**Country:** United Kingdom<br><br>**<u>Participants</u>**<br>**Recruitment:** No details reported<br>**Age:** 22<br>**Sex:** 20 F, 20 M<br>**Total number of participants:** 40<br>**Dropouts:** 4<br>**Reason for drop out:** Failed to produce sufficient saliva for analysis<br>**Revised sample size:** 36<br>**Analgesia intake:** None<br>**Chronic conditions:** Healthy (psychiatric conditions and chronic pain not

		specifically reported)<br>**Restrictions:** Alcohol, vigorous exercise, food, caffeine<br><br>**Study design:**<br>Salivary sIgA measured before and after an induced noxious stimulus (first and second exposures in different arms)<br>**Other measurements:** Blood pressure and heart rate, pain intensity<br>**Intervention:** Cold pain induced by CPT<br>**Comparison:** Change from baseline. Changes compared between first and second exposures.<br>**Salivary assay:** Radial immunodiffusion (RID) assay (Bind A Rid, The Binding Site Ltd, Winzer 1999)<br>**Saliva type:** Stimulated saliva<br>**Collection method:** Swab<br><br>**OUTCOMES**<br>sIgA levels fell significantly after first exposure to CPT, but not after second exposure in the same participants' other arm<br>**Correlation with pain ratings:** No significant difference found<br>**Sex effects:** No significant difference found
19	Christidis (2020) Alpha-amylase	**AIM**<br>To investigate whether the levels of sAA are influenced by experimentally induced muscle pain<br><br>**METHOD**<br>**Country:** Sweden<br><br>**Participants**<br>**Recruitment:** Advertisement on social media and among undergraduate dental students at Karolinska Institute<br>**Age:** 23.8<br>**Sex:** 13 F, 13 M<br>**Total number of participants:** 26<br>**Dropouts:** 0<br>**Reason for drop out:** Not applicable<br>**Revised sample size:** 26<br>**Analgesia intake:** None<br>**Chronic conditions:** Healthy (psychiatric conditions not specifically reported, chronic pain part of exclusion criteria)<br>**Restrictions:** All of food, alcohol, smoking, caffeine and precautions to reduce risk of contamination due to bleeding from gums<br><br>**Study design:**<br>Salivary AA measured before and after an induced somatic noxious stimulus<br>**Other measurements:** Depression and anxiety, somatic symptoms, pain

		intensity<br>**Interventions:** Hypertonic saline muscle injection<br>**Comparison**: Change from baseline<br>**Salivary assay:** Commercially available enzymatic assay kit<br>**Saliva type:** Stimulated saliva<br>**Collection method:** Passive drool<br><br>**OUTCOMES**<br>No change in sAA<br>**Pain intensity:** Not analysed<br>**Sex differences:** No difference
20	Cruz-Almeida (2017)<br>Panel of cytokines (IL-6, IL-8, IL-10, IL-4)	**AIM**<br>To characterize the time course, duration and magnitude of changes of commonly measured pro- (interleukin [IL]-6, IL-8) and anti-inflammatory (IL-10, IL-4) cytokines in saliva samples and to test for age-related differences<br><br>**METHODS**<br>**Country:** United States of America<br><br>**Participants**<br>**Recruitment:** Details not reported<br>**Age:** 8 younger participants: mean 21.4; 9 older participants: mean age 68.1<br>**Sex:** 8 F, 9 M<br>**Total number of participants:** 17<br>**Dropouts:** 1<br>**Reason for drop out:** Vigorous physical activity before arriving for the session<br>**Revised sample size:** 16<br>**Analgesia intake:** None<br>**Chronic conditions:** Healthy (chronic pain and psychiatric conditions part of exclusion criteria)<br>**Restrictions:** All of food, alcohol, smoking, caffeine<br><br>**Study design**<br>A panel of salivary cytokines (IL-6, IL-8, IL-10, IL-4) measured before and after an induced noxious stimulus or a non-noxious control in saliva and blood<br>Non-painful task was done on the same participants with and without venepuncture<br>**Other measurements:** Blood pressure, pain intensity,<br>**Intervention:** Cold pain induced by CPT with a non-painful thermal water task as control<br>**Salivary Assay:** MILLIPLEX XMAP human cytokine/chemokine-premixed 13-Plex assay

		**Comparisons:** Change from baseline. Changes compared (1) between the CPT and control groups, (2) between the two age groups, (3) with or without venipuncture in control group<br>**Saliva type:** Unstimulated saliva<br>**Salivary collection method:** Swab<br><br>**<u>OUTCOMES</u>**<br>IL-6, IL-10 & IL-4 concentrations increased from baseline, peaking at 60 minutes after CPT<br>IL-8 peaked at 45 minutes after CPT<br>No significant changes reported in control group<br>Venepunture had no significant effect on the cytokine levels<br>**Correlation with pain ratings:** Not analysed<br>**Sex effects:** Not analysed<br><br>**<u>NOTES</u>**<br>The time course of the peak levels of cytokines in the CPT session was nearly identical in saliva and plasma<br>Older adults experienced greater salivary changes in all cytokines during the cold pressor session compared to younger adults in the non-painful sessions
21	Finke (2021) Cortisol	**<u>AIM</u>**<br>To assess how concurrent administration of a cognitive and physical stressor affects stress response patterns on subjective and physiological dimensions<br><br>**<u>METHOD</u>**<br>**Country:** Germany<br><br>**<u>Participants</u>**<br>**Recruitment:** University's email newsletter<br>**Age:** Mean 23<br>**Sex:** 28 F, 28 M<br>**Total number of participants:** 56<br>**Dropouts:**<br>From CPT: 0. From cognitive stress task: 0. From cardiovascular parameters heart rate variation data analysis: 2. From respiration breathing pattern analysis: 3. From saliva sampling: 0, From pain intensity rating: 1, From voice frequency analysis 3,<br><br>**Reason for drop out:** Artefacts in ECG (2), poor quality respiratory data (3), technical failure in recording subjective rating (1), technical failure in speech recording (3)<br>**Revised sample size:** 56<br>**Analgesia intake:** Occasional use of simple analgesics allowed<br>**Chronic conditions:** Healthy (psychiatric conditions and chronic pain part

		of exclusion criteria)<br>**Restrictions:** All of food, alcohol, smoking, caffeine<br><br>**<u>Study design:</u>**<br>Four conditions (fully crossed interventions, evenly divided across sexes): (i) CP with simultaneous PASAT; (ii) CP without PASAT; (iii) PASAT during warm-water exposure; (iv) warmwater procedure without PASAT (control)<br><br>**Salivary assay:** Time-resolved immunoassay with fluorescence detection<br>**Saliva type:** Unclear<br>**Salivary collection method:** Swab<br><br>**<u>OUTCOMES</u>**<br>Intervention: Cold pain induction or a warm-water control condition<br>In half of the sample, the Paced Auditory Serial Addition Task (PASAT) was performed simultaneously (fully crossed interventions). Salivary cortisol, cardiovascular parameters, and subjective ratings as well as voice pitch (F0) were assessed<br>Comparison with<br>**Correlation with pain ratings:** Not analysed<br>**Sex effects:** Not analysed<br><br>**<u>NOTES</u>**<br>**Inclusion in the cortisol- cold pain quantitative analysis:** Yes<br><br>**<u>BIAS</u>**<br>**Bias Type:** Moderate in selection of participants and selection of reported results<br>**Author's judgement:** Moderate ROB<br>**Support for judgement:** Recruitment limited to the university community, no published pre-specified protocol
22	Gaab (2016) sAA, cortisol	**<u>AIM</u>**<br>To investigate the effects of acute psychosocial stress on heat pain perception and salivary cortisol levels and α-amylase activity<br><br>**<u>METHODS</u>**<br>**Country:** Germany<br><br>**<u>Participants</u>**<br>**Recruitment:** E-mail or oral promotion in lectures of the Department of Psychology, University of Basel<br>**Age:** Mean 24.6<br>**Gender:** Male only<br>**Total number of participants:** 29<br>**Dropouts:** 0<br>**Reason for drop out:** Not applicable

		**Revised sample size:** 29<br>**Analgesia intake:** None<br>**Chronic conditions:** Healthy (psychiatric conditions and chronic pain part of exclusion criteria)<br>**Restrictions:** Smoking, alcohol<br><br>**<u>Study design:</u>**<br>Salivary sAA and cortisol measured before and after exposure to noxious heat first before and after the Trier social stress test compared with a control condition with cross over design<br>**Other measurements:** Pain intensity, anxiety<br>**Interventions:** Acute heat pain induced using the Peltier device<br>**Comparison:** Change in cortisol and sAA from baseline before and after heat pain. Comparison of change before and after stress test.<br>**Salivary assay:** Cortisol: Highly sensitive liquid chromatography-tandem mass spectrometry. sAA: kinetic colorimetric test assay<br>**Saliva type:** Unstimulated<br>**Salivary collection method:** Swab<br><br>**<u>OUTCOMES</u>**<br>Heat pain alone was not associated with significant change in sAA and cortisol, while psychosocial stress was<br>**Correlation with pain ratings:** No significant correlation for cortisol<br>**Sex effects:** Not analysed<br><br>**<u>NOTES</u>**<br>The pattern of cortisol change was similar to sAA
23	Geiss (2012)<br>IL-6<br>Cortisol	**<u>AIM:</u>**<br>To investigate the pathophysiologic relevance of cortisol levels for manifestation of fibromyalgia syndrome<br><br>**<u>METHODS</u>**<br>**Country:** Germany<br><br>**<u>Participants</u>**<br>**Recruitment:** Fibromyalgia patients from a local support group. Pain-free participants matched for age and education from local newspaper and bulletin board advertisements.<br>**Age:** Fibromyalgia patients: mean 50, Controls: mean 41<br>**Sex:** Female only<br>**Total number of participants:** 27 (12 with fibromyalgia, 15 healthy controls)<br>**Dropouts:** 0<br>**Reason for drop out:** Not applicable<br>**Revised sample size:** 27<br>**Analgesia intake:** None

		**Chronic conditions:** Psychiatric conditions part of exclusion criteria.<br>**Restrictions:** Food, exercise<br><br>**<u>Study design</u>**<br>Salivary IL-6 and cortisol measured before and after an induced noxious stimulus<br>**Other measurements:** Blood white cell count, free cortisol, ACTH, catecholamines, IL-6, pain intensity, fatigue levels, chronic stress levels<br>**Intervention:** Mechanical pressure pain thresholds induced by algometry in 8 defined anatomical points<br>**Saliva Assays:** IL-6: ELISA. Cortisol: time resolved fluorescence immunoassay<br>**Comparisons:** Change from baseline. Change compared between patients with fibromyalgia and healthy pain free women.<br>**Saliva Type:** Unclear if stimulated or unstimulated<br>**Salivary collection method:** Swab<br><br>**<u>OUTCOMES</u>**<br>In women with fibromyalgia, IL-6 and cortisol increased significantly 10 minutes after measuring pain pressure thresholds but this did not happen in the healthy subjects<br>**Correlation with pain ratings:** Not analysed<br>**Sex effects:** Not applicable (female only study)
24	Geva (2014)<br>Cortisol	**<u>AIM</u>**<br>To explore the effects of acute stress on pain perception, pain intensity and the associated stress response<br><br>**<u>METHOD</u>**<br>**Country:** Israel<br><br>**<u>Participants</u>**<br>**Recruitment:** Advertisements posted at the university<br>**Age:** 33<br>**Sex:** Male only<br>**Total number of participants:** 29<br>**Dropouts:** 0<br>**Reason for drop out:** Not applicable<br>**Revised sample size:** 29<br>**Analgesia intake:** Not reported<br>**Chronic conditions:** Healthy (psychiatric and chronic pain part of exclusion criteria)<br>**Restrictions:** Exercise, food, caffeine<br><br>**<u>Study design:</u>**<br>Salivary cortisol measured before and after exposure to induced noxious heat twice; before and during the Montreal Imaging Stress Task (MIST)

		**Other measurements:** Perceived stress, anxiety, heart rate, blood pressure, respiratory rate, skin conductance, pain intensity<br>**Interventions:** Heat pain using Peltier device<br>**Comparison:** Change from baseline before and after two noxious heat stimulus sessions performed on either side of stress manipulation<br>**Salivary assays:** Commercial ELISA<br>**Saliva type:** Unstimulated<br>**Salivary collection method:** Swab<br><br>**<u>OUTCOMES</u>**<br>Change in salivary cortisol after heat pain was not significant before the stress task. In contrast, cortisol levels increased significantly in response to heat pain after the participants had done the stress task.<br>In contrast, cortisol levels increased significantly in response to heat pain after the participants had done the stress task.<br>**Correlation with pain ratings:** Not analysed<br>**Sex effects:** Not analysed
25	Geva (2017)<br>Cortisol	**<u>AIM</u>**<br>To test pain inhibition capabilities of triathletes under acute, controlled psychological stress manipulation<br><br>**<u>METHOD</u>**<br>**Country:** Israel<br><br>**<u>Participants</u>**<br>**Recruitment:** Advertisements posted at the university and internet sites of triathlon<br>**Age:** Mean 35.9<br>**Gender:** Male<br>**Total number of participants:** 25<br>**Dropouts:** 0<br>**Reason for drop out:** Not applicable<br>**Revised sample size:** 25<br>**Analgesia intake:** Not reported<br>**Chronic conditions:** Healthy (psychiatric conditions and chronic pain part of exclusion criteria)<br>**Restrictions:** Exercise, food, caffeine<br><br>**<u>Study design:</u>**<br>Measurement of salivary cortisol before and after noxious heat stimulus twice; before and after the application of the stress manipulation (using the Montreal Imaging Stress Task)<br>**Other measurements:** Skin conductance, perceived stress, anxiety, heart rate and heart rate variation, pain intensity<br>**Interventions:** Acute heat pain induced by Peltier-based computerized thermal stimulators

		**Comparison:** Change from baseline before and after two noxious heat sessions performed on either side of stress manipulation<br>**Salivary assay:** ELISA<br>**Saliva type:** Unstimulated<br>**Salivary collection method:** Swab<br><br>**<u>OUTCOMES</u>**<br>Change in salivary cortisol after heat pain was not significant before the stress task. In contrast, cortisol levels increased significantly in response to heat pain after the participants had done the stress task<br>In contrast, cortisol levels increased significantly in response to heat pain after the participants had done the stress task.<br>**Correlation with pain ratings:** Not analysed<br>**Sex effects:** Not analysed
26	Geva (2018)<br>Cortisol	**<u>AIM</u>**<br>To study the effect of acute psychosocial stress manipulation on pain modulation<br><br>**<u>METHOD</u>**<br>**Country:** Israel<br><br>**<u>Participants</u>**<br>**Recruitment:** By advertisements posted around the university campus<br>**Age:** Mean 34<br>**Gender:** Male only<br>**Total number of participants:** 31<br>**Dropouts:** 0<br>**Reason for drop out:** Not applicable<br>**Revised sample size:** 31<br>**Analgesia intake:** Not reported<br>**Chronic conditions:** Healthy (psychiatric conditions and chronic pain part of exclusion criteria)<br>**Restrictions:** Exercise, food, caffeine<br><br>**<u>Study design:</u>**<br>Measurement of salivary cortisol after heat pain induction twice; before and after the application of the stress manipulation (using the Montreal Imaging Stress Task)<br>**Other measurements:** Skin conductance, anxiety, heart rate and heart rate variation, blood pressure, respiration, heart rate variability, perceived stress, pain intensity<br>**Comparison:** Change from baseline before and after two testing sessions performed on either side of stress manipulation<br>**Interventions:** Heat pain using Peltier thermal stimulator<br>**Salivary assay:** Cortisol: ELISA<br>**Saliva type:** Unstimulated

		**Salivary collection type:** Swab<br><br>**<u>OUTCOME</u>**<br>Change in salivary cortisol after heat pain was not significant before the stress task. In contrast, cortisol levels increased significantly in response to heat pain after the participants had done the stress task.<br>**Correlation with pain ratings:** Not analysed<br>**Sex effects:** Not analysed
27	Geva (2022) Cortisol	**<u>AIM</u>**<br>To examine the effects of psychosocial stress on pain perception and modulation of women and men<br><br>**<u>METHOD</u>**<br>**Country:** Israel<br>**Aim:** To examine the effect of psychosocial stress on pain perception and modulation<br><br>**<u>Participants</u>**<br>**Recruitment:** Recruited by advertisements posted around the university campus<br>**Age:** F: Mean 30.9, M: Mean 28.3<br>**Sex:** F 82, M 66<br>**Total number of participants:** 148<br>**Dropouts:** 0<br>**Reasons for drop out:** Not applicable<br>**Revised sample size:** 148<br>**Analgesia intake:** None<br>**Chronic conditions:** Healthy (psychiatric conditions and chronic pain part of exclusion criteria)<br>**Restrictions:** Food, caffeine, exercise<br><br>**<u>Study design:</u>**<br>Salivary cortisol measured before and after induced noxious heat stimulus followed by MIST (n=133) or sham task (n=15) and then also after a second episode of induced noxious stimulation.<br>**Other measurements:** Pain intensity, heart rate, heart rate variability, galvanic skin response<br>**Interventions:** Heat pain using Peltier-based computerized thermal stimulators<br>**Comparison:** Change in cortisol from baseline<br>**Salivary assay:** ELISA<br>**Saliva type:** Stimulated<br>**Salivary Collection method:** Swab<br><br>**<u>OUTCOMES</u>**<br>There was no change in salivary cortisol in the sham group

		**Correlation with pain ratings:** Not reported in the sham group<br>**Sex effects:** No difference in the sham group<br><br>**NOTES**<br>In participants who were exposed to MIST, cortisol levels increased in men and fell back down during the recovery phase. In women this increase did not reach significance. Among men, temporal summation of pain increased following the MIST but was not predicted by the stress variables. The authors concluded that acute stress manipulation affects stress and pain responses in women and men differently: women exhibited stress-induced anti-nociception and men exhibited stress-induced pro-nociception.
29	Goodin (2012)-1 sTNFαR-II, Cortisol	**AIM**<br>To characterize the neuroendocrine and inflammatory responses to multiple experimental pain modalities<br><br>**METHODS**<br>**Country:** United States of America<br><br>**Participants**<br>**Recruitment:** College students recruited, details not reported<br>**Age:** 20.2<br>**Sex:** F 24, M 22<br>**Total number of participants:** 46<br>**Dropouts:** 0<br>**Reason for drop out:** Not applicable<br>**Revised sample size:** 46<br>**Analgesia intake:** None<br>**Chronic conditions:** Healthy (chronic pain and psychiatric conditions part of exclusion criteria)<br>**Restrictions:** All food, alcohol, smoking, caffeine<br><br>**Study design**<br>Salivary sTNFαR-II and cortisol measured before and after an induced noxious stimulus<br>**Other measurements:** Pain intensity, pain unpleasantness<br>**Intervention:** Exposure to multiple pain modalities (cold, heat and ischaemic pain) induced by CPT, HWT IPT or room temperature water (control)<br>**Comparisons:** Change from baseline. Changes compared between the painful pain modalities and the control group<br>**Salivary assays:** Cortisol: High sensitivity immunoassay. sTNFαRII: Human sTNFαRII enzyme immunoassay<br>**Saliva type:** Stimulated saliva<br>**Salivary collection method:** Swab

		<u>**OUTCOMES**</u><br>Cortisol: Cold pain but not heat or ischaemic pain produced significant time-dependent elevation, whereas cortisol significantly decreased for the neutral water task<br>sTNFαRII: The cold pressor, hot water, and ischemic modalities were associated with significant reduction over time, especially 25-35 minutes after pain induction. Response to neutral water initially decreased but returned to approximate baseline.<br>**Correlation with pain ratings:** Significant positive correlation between cortisol change from baseline and pain intensity ratings<br>**Sex effects:** Not analysed<br><br><u>**NOTES**</u><br>Researchers were aiming to assess salivary pro-inflammatory cytokines after acute pain induction and chose to measure sTNFαR-II, because it is more stable than TNFα and can be measured more reliably<br>Cortisol response was negatively associated with the overall sTNFαRII response<br>**Inclusion in cortisol-cold pain quantitative analysis:** Yes, for healthy participants (n=10)<br><br><u>**RISK OF BIAS FOR CORTISOL-COLD PAIN ANALYSIS**</u><br>**Bias Type:** Moderate for risks of confounding, selection of participants, and departures from intended exposure and measurement of outcome<br>**Author's judgement:** Moderate risk of bias<br>**Support for judgement:** No control but steps taken to reduce stress and anxiety in participants, recruitment limited to university community, interactions between participants and experimenters not fully described and no published pre-specified protocol.
30	Goodin (2012)-2<br>sTNFαR-II<br>Cortisol	<u>**AIM**</u><br>To examine the association between cortisol awakening response (CAR) and acute pain stimulation and whether CAR was related with salivary cortisol and soluble tumour necrosis factor-α receptor II (sTNFαRII) responses to acute pain induction<br><br><u>**METHOD**</u><br>**Country:** United States of America<br><br><u>**Participants**</u><br>**Recruitment:** Recruited from an urban university setting, no further details<br>**Age:** Mean 36<br>**Sex:** 17 F, 19 M<br>**Total number of participants:** 36<br>**Dropouts:** 0<br>**Reason for drop out:** Not applicable

		**Revised sample size:** 36<br>**Analgesia intake:** None<br>**Chronic conditions:** Healthy (chronic pain and psychiatric conditions part of exclusion criteria)<br>**Restrictions:** All of food, alcohol, smoking, caffeine<br><br>**<u>Study design:</u>**<br>Salivary sTNFαR-II and cortisol measured before and after noxious stimuli<br>**Other measurements:** Pain intensity, perceived stress, morning salivary cortisol levels for CAR,<br>**Interventions:** Exposure to multiple pain modalities (cold, heat, ischaemic pain induced by CPT, hot water task and ischaemic pain task<br>**Comparisons:** Change from baseline<br>**Salivary assays:** Cortisol: High sensitivity salivary cortisol immunoassay. sTNFαRII: Human sTNFαRII enzyme immunoassay<br>**Saliva type:** Stimulated saliva<br>**Collection method:** Swab<br><br>**<u>OUTCOMES</u>**<br>sTNFαR-II: Significant reduction immediately after pain induction<br>Cortisol: Significant elevation after pain induction<br>**Correlation with pain ratings:** Not analysed<br>**Sex effects:** Not analysed<br><br>**<u>NOTES</u>**<br>Researchers were aiming to assess salivary pro-inflammatory cytokines after acute pain induction and chose to measure sTNFαR-II, because it is more stable than TNFα and can be measured more reliably<br>**Inclusion in cortisol-cold pain quantitative analysis:** No. Reason: Results were not separated for different pain modalities
28	Goodin (2012)-3 Cortisol	**<u>AIM</u>**<br>To investigate the effect of sleep quality on pain intensity and cortisol reactivity<br><br>**<u>METHOD</u>**<br>**Country:** United States of America<br><br>**<u>Participants</u>**<br>**Recruitment:** Recruited from a college campus using posted advertisements<br>**Age:** Mean 20.2<br>**Sex:** 20 F, 20 M<br>**Total number of participants:** 40<br>**Dropouts:** 0<br>**Reason for drop out:** Not applicable<br>**Revised sample size:** 40

		**Analgesia intake:** None<br>**Chronic conditions:** Healthy (psychiatric conditions and chronic pain part of exclusion criteria)<br>**Restrictions:** Food, alcohol, smoking, caffeine. Precautions to reduce risk of contamination due to bleeding from gums<br><br>**<u>Study design</u>**<br>Salivary cortisol measured before and after an induced noxious stimulus<br>**Other measurements:** Sleep quality, pain intensity, pain characteristics, affect<br>**Intervention:** Cold pain induced using CPT<br>**Comparison:** Change from baseline<br>**Salivary assay:** High sensitivity immunoassay kits<br>**Saliva type:** Stimulated<br>**Salivary collection method:** Swab<br><br>**<u>OUTCOMES</u>**<br>Poor sleep quality was significantly associated with greater reports of CPT-induced pain severity and greater cortisol increase from baseline<br>**Correlation with pain ratings:** Significant positive correlation<br>**Sex effects:** No significant difference<br><br>**<u>NOTES</u>**<br>**Inclusion in the cortisol- cold pain quantitative analysis:** Yes<br><br>**<u>BIAS</u>**<br>**Bias Type:** Moderate for risks of confounding, selection of participants, and departures from intended exposure and measurement of outcome<br>**Author's judgement:**<br>**Support for judgement:** No control but steps taken to minimise participant stress, recruitment from the university community, interactions between participants and experimenter not fully described, no published pre-specified protocol
31	Hengesch (2018) Cortisol	**<u>AIM</u>**<br>To investigate the association of exposure to early life adversity (ELA) and adult stress reactivity<br><br>**<u>METHOD</u>**<br>**Country:** Luxembourg<br><br>**<u>Participants</u>**<br>**Recruitment:** From Luxembourg and the greater region Saar-Lor-Lux<br>**Age:** Controls: 21.8. Participants with ELA: 22.5<br>**Sex:** Healthy participants: 11 F, 11 M. ELA participants: 14 F, 8 M<br>**Total number of participants:** 44 (22 healthy and 22 ELA)<br>**Dropouts:** 0

		**Reason for drop out:** Not applicable<br>**Revised sample size:** 44<br>**Analgesia intake:** Yes<br>**Chronic conditions:** ELA or healthy (psychiatric conditions and chronic pain not specifically recorded in either group)<br><br>**<u>Study design:</u>**<br>Salivary cortisol measured before and after an induced noxious cold stimulus combined with a stressful cognitive task in people with ELA and healthy matched controls<br>**Other measurements:** Stress, arousal, anxiety, pain intensity, heart rate, blood pressure<br>**Interventions:** Acute cold pain using CPT combined with stress using the Paced Auditory Serial Addition Task (PASAT)<br>**Comparison:** Change from baseline. Comparison between change in those with ELA and the control group<br>**Salivary assay:** Time resolved immunoassay with fluorescence detection<br>**Saliva type:** Unclear<br>**Collection method:** Swab<br><br>**<u>OUTCOMES</u>**<br>In people with early life adversity (ELA) the cortisol response after CPT combined with PASAT was blunted compared with people who had not experienced ELA (even though there was no difference in reported pain intensity between the groups)<br>**Correlation with pain ratings:** Not analysed<br>**Sex effects:** No significant difference<br><br>**<u>NOTES</u>**<br>Inclusion in the cortisol- cold pain quantitative analysis: No<br>**Reason:** Experimental design included stress induced by a cognitive task as well as noxious stimulus for participants
32	Hoeger-Bement (2010) Cortisol	**<u>AIM</u>**<br>To investigate the influence of exposure to a cognitive stressor on pain perception and determine the individual characteristics that may be predictors of the pain response<br><br>We examined pain perception to a mechanical noxious stimulus before and after exposure to a cognitive stressor across a range of pain responses. Mental math was used as the cognitive stressor because it is an established and effective psychosocial technique to induce stress [1]. Changes in stress and anxiety were assessed with both self-reported and physiological measures including questionnaires, visual analogue scales (VAS), mean arterial pressure (MAP), heart rate, and salivary cortisol levels.

		**METHOD**<br>**Country:** United States of America<br><br>**Participants**<br>**Recruitment:** No details<br>**Age:** 20.2<br>**Sex:** 13 F, 12 M<br>**Total number of participants:** 25<br>**Dropouts:** 0<br>**Reason for drop out:** Not applicable<br>**Revised sample size:** 25<br>**Analgesia intake:** Not reported<br>**Chronic conditions:** Healthy (psychiatric conditions part of exclusion criteria, chronic pain not reported)<br>**Restrictions:** Food, alcohol, smoking, mouth<br><br>**Study design:**<br>Salivary cortisol measured before and after two induced mechanical pressure noxious stimulus tests that were done on either side of 1) a mental math task (stressor) and 2) a rest (control) session in a cross over design<br>**Other measurements:** Pain intensity, blood pressure, heart rate, state anxiety, perceived stress<br>**Interventions:** Acute pressure pain induced using a pain pressure device<br>**Comparison:** Change from baseline after pain tests compared between the stressor session and the rest session<br>**Salivary assay:** Enzymatic immune-assay<br>**Saliva type:** Stimulated<br>**Salivary collection method:** Salivary Swab<br><br>**OUTCOMES**<br>Salivary cortisol did not change from baseline when the participants did not do the stressor task. There was significant rise in cortisol after pain induction when participants were due to do the stress task.<br>**Correlation with pain ratings:** Not analysed<br>**Sex effects:** No significant difference<br><br>**NOTES**<br>The authors concluded that rise in cortisol is related to anticipation of the stressor and not to pressure pain induction
33	Icenhour (2020) Cortisol	**AIM**<br>To elucidate the role of chronic stress in visceral nociception<br><br>**METHOD**<br>**Country:** Germany

		**<u>Participants</u>**<br>**Recruitment:** Local advertisements<br>**Age:** Mean 26.38<br>**Sex:** 90 F, 90 M<br>**Total number of participants:** 180 (tertiles based on Trier Inventory for Chronic Stress: 61 high stress, 57 low stress)<br>**Dropouts:** 62 in the mid-tertile not included in analysis<br>**Reason for drop out:** Not applicable<br>**Revised sample size:** 118<br>**Analgesia intake:** None<br>**Chronic conditions:** Healthy (psychiatric conditions part of exclusion criteria, chronic pain not specifically reported)<br>**Restrictions:** None<br><br>**<u>Study design:</u>**<br>Differences in response to noxious visceral stimulus compared between participant groups of elevated perceived chronic stress and low perceived chronic stress<br>**Other measurements:** Pain intensity, state anxiety, general self-efficacy<br>**Intervention:** Acute visceral pain induced by balloon rectal distensions<br>**Comparison:** Change from baseline compared between high and low chronic stress groups<br>**Salivary assay:** Cortisol: ELISA<br>**Saliva type:** Unclear<br>**Collection method:** Swab<br><br>**<u>OUTCOMES</u>**<br>Cortisol levels were significantly higher throughout the experiment in those with higher perceived stress but there was no rise in cortisol in either group on measuring visceral pain thresholds<br>**Correlation with pain ratings:** Not analysed<br>**Sex effects:** Not analysed<br><br>**<u>NOTES</u>**<br>Significantly elevated state anxiety and cortisol concentrations were observed in the cohort with higher perceived chronic stress across experimental time points
53	Inayama (2022) Alpha-amylase	**<u>AIM</u>**<br>To examine the hypothesis that listening to music decreases the pain of vascular access cannulation for haemodialysis<br><br>**<u>METHOD</u>**<br>**Country:** Japan<br><br>**<u>Participants</u>**<br>**Recruitment:** Dialysis patients in 5 centres who reported cannulation pain

		in a preliminary questionnaire<br>**Age:** median 64 (mean not stated)<br>**Sex:** F 35, M 86<br>**Total number of participants:** 121<br>**Dropouts:** 4<br>**Reasons for drop out:** patient withdrawal (4), protocol violations (17)<br>**Revised sample size:** 99<br>**Analgesia intake:** Not reported<br>**Chronic conditions:** Renal impairment needing regular dialysis (psychiatric conditions and chronic pain not reported)<br>**Restrictions:** none reported<br><br>**<u>Study design:</u>**<br>Salivary amylase measured before and after vascular cannulation during classical music intervention compared to a white noise control group in a cross over, single blind, randomized trial<br>**Other measurements:** pain intensity, anxiety, blood pressure<br>**Interventions:** Painful cannulation for haemodialysis vascular access<br>**Comparison:** Difference between the intervention and control groups<br>**Salivary assay:** Not stated<br>**Saliva type:** Not stated<br>**Salivary collection method:** Unknown<br><br>**<u>OUTCOMES</u>**<br>There were no significant differences in salivary amylase<br>**Correlation with pain ratings:** Not analysed<br>**Sex effects:** Not analysed
34	Larra (2015)<br>Alpha-amylase<br>Cortisol	**<u>AIM:</u>**<br>To compare the neuroendocrine stress response elicited by bilateral feet CPT and the classical dominant hand CPT<br><br>**<u>METHODS</u>**<br>**Country:** Germany<br><br>**<u>Participants</u>**<br>**Recruitment:** Details not reported<br>**Age:** Mean age 22.5<br>**Sex:** 12 F, 12 M<br>**Total number of participants:** 24<br>**Dropouts:** 2<br>**Reason for drop out:** CPT terminated prematurely (1), baseline saliva sample could not be analysed (1)<br>**Revised sample size:** 23 for sAA, 22 for cortisol analysis<br>**Analgesia intake:** None<br>**Chronic conditions:** Healthy (psychiatric conditions part of exclusion criteria, chronic pain not specifically reported)

		**Restrictions:** Food, alcohol, smoking, caffeine, precautions to reduce risk of contamination due to bleeding from gums<br><br>**<u>Study design:</u>**<br>Salivary cortisol and sAA measured before and after an induced noxious cold stimulus to hand and then separately to both feet in a crossover design<br>**Other measurements:** Heart rate, blood pressure, stress levels, pain intensity<br>**Comparison:** Change from baseline compared between hand and feet CPT<br>**Interventions:** Acute cold pain induced by CPT<br>**Salivary assays:** Cortisol: time-resolved immunoassay with fluorescence detection. sAA: Quantitative enzyme kinetic method<br>**Saliva type:** Stimulated saliva<br>**Collection method:** Spitting<br><br>**<u>OUTCOMES</u>**<br>sAA: Significant rise after both feet and hand CPT<br>Cortisol: Decreased after hand CPT but increased after foot CPT<br>**Correlation with pain ratings:** Not analysed<br>**Sex effects:** No significant differences found for cortisol or sAA<br><br>**<u>NOTES</u>**<br>**Inclusion in the cortisol- cold pain quantitative analysis:** Yes<br>Hand CPT experiment and feet CPT experiment entered in quantitative analysis as 2 separate experiments<br><br>**<u>BIAS</u>**<br>**Bias Type:** High in selection of participants. Moderate in confounding, missing data and selection of reported result.<br>**Author's judgement:** High ROB<br>**Support for judgement:** Method of participant recruitment not reported. No neutral control but steps taken to minimise participant stress. Missing data excluded from all analysis and not accounted for but no indication of differential loss related to prognostic factors. No published pre-specified protocol.
52	Lorenz (2021) Cortisol	**<u>AIM</u>**<br>To compare the physical and psychological stress responses to finger prick and venepuncture<br><br>**<u>METHOD</u>**<br>**Country:** United States of America<br><br>**<u>Participants</u>**<br>**Recruitment:** Flyers and e-mail announcements to the university list reserve and psychology participant pool

		**Age:** Mean 21.93<br>**Sex:** F only<br>**Total number of participants:** 45<br>**Dropouts:** 5<br>**Reasons for drop out:** Did not complete both experimental sessions<br>**Revised sample size:** 40<br>**Analgesia intake:** Not reported<br>**Chronic conditions:** Healthy (psychiatric conditions and chronic pain not specifically recorded)<br>**Restrictions:** food, alcohol, smoking<br><br>**<u>Study design:</u>**<br>Measurement of salivary cortisol before and after finger prick or venepuncture with a cross over design<br>**Other measurements:** Pain intensity, heart rate, heart rate variability, stress, affect,<br>**Interventions:** Acute pain after drawing blood by venepuncture or by finger prick<br>**Comparison:** Change from baseline with comparison between the 2 procedures.<br>**Salivary assay:** ELISA kits<br>**Saliva type** Unstimulated<br>**Salivary Collection method** Passive drool<br><br>**<u>OUTCOMES</u>**<br>Significant decline in cortisol at 10 and 20 minutes after drawing blood with venepuncture but no change with finger prick<br>**Correlation with pain ratings:** No correlation<br>**Sex effects:** Not applicable (all F)<br><br>**<u>NOTES</u>**<br>Psychological measures of stress such as negative emotion and perceived stress, were stronger predictors of reported pain than physical stress measures such as blood pressure and heart rate<br>Pre-procedure mean cortisol levels were at the high end of the normal range for sex and time of day regardless of which procedure was being done. The authors concluded that anticipatory anxiety leads to cortisol rise that subsides after the event.
35	Lukacs (2022) Cortisol	**<u>AIM</u>**<br>To examine the relationship between conditioned pain modulation and SNS and HPA reactivity where pressure pain was studied before and after CPT<br><br>**<u>METHOD</u>**<br>**Country:** Canada

		<u>**Participants**</u><br>**Recruitment:** university-level participants, purposive recruitment to ensure equal representation of sexes<br>**Age:** 24.5<br>**Gender:** 25 F, 25 M<br>**Total number of participants:** 50<br>**Dropouts:** 0<br>**Reason for drop out:** Not applicable<br>**Revised sample size:** 50<br>**Analgesia intake:** None<br>**Chronic conditions:** Healthy (psychiatric conditions not specifically reported, chronic pain part of exclusion criteria)<br>**Restrictions:** food, alcohol, exercise<br><br><u>**Study design:**</u><br>Salivary cortisol measured before and after an induced noxious cold stimulus<br>**Other measurements:** Pain intensity, galvanic skin response<br>**Interventions:** Acute cold pain induced by CPT as the noxious conditioning stimulus. Pressure pain detection threshold measured before and after CPT.<br>**Comparison:** Change from baseline<br>**Salivary assay:** ELISA<br>**Saliva type** Unstimulated<br>**Salivary Collection method** Swab<br><br><u>**OUTCOMES**</u><br>No significant change in salivary cortisol 30 seconds after exposure to noxious cold<br>**Correlation with pain ratings:** Not analysed<br>**Sex effects:** Not analysed<br><br><u>**NOTES**</u><br>**Inclusion in the cortisol- cold pain quantitative analysis:** Yes
36	Meeus (2009) Cortisol	<u>**AIM**</u><br>To evaluate endogenous pain inhibition and the cortisol response in chronic fatigue syndrome patients with chronic widespread pain compared with a healthy control group using spatial summation of thermal noxious stimuli<br><br><u>**METHODS**</u><br>**Country:** Belgium<br><br><u>**Participants**</u><br>**Recruitment:** Study patients: Random selection from the medical files available at the university-based chronic fatigue clinic. Control subjects:

		From the staff and students of the university physiotherapy department and among friends and family of the researchers (age and gender-matched)<br>**Age:** Mean: 44.4<br>**Sex:** CSF: 21 F, 10 M**.** Healthy: 21 F, 10 M<br>**Total number of participants:** 62 (31 CFS-patients with chronic pain, 31 controls)<br>**Dropouts:** 0<br>**Reason for drop out:** Not applicable<br>**Revised sample size:** 62<br>**Analgesia intake:** Not reported<br>**Chronic conditions:** Healthy control group (psychiatric conditions not specifically recorded, chronic pain excluded in control group)<br>**Restrictions:** Exertion, caffeine, alcohol, smoking<br><br>**Study design**<br>Measurement of salivary cortisol before and after induced noxious heat stimulus in people with chronic fatigue syndrome and widespread pain compared with controls<br>**Other measurements:** Pain intensity,<br>**Interventions:** Acute heat pain induced by hot water immersion<br>**Comparison:** Change from baseline compared in people with chronic fatigue and widespread pain and healthy controls<br>**Salivary assay:** Radioimmunoassay<br>**Saliva type:** Unclear<br>**Salivary collection method:** Swab<br>**Restrictions:** Exertion, caffeine, alcohol, smoking<br><br>**OUTCOMES**<br>No significant change in salivary cortisol either group<br>**Correlation with pain ratings:** In people with chronic fatigue syndrome (CFS) there was significant negative correlation between change in cortisol and pain intensity<br>**Sex effects:** Not analysed
55	Muhtz (2013) Cortisol	**AIM**<br>To examine the effects of pain stimuli on cortisol levels in patients with chronic pain and patients with depression<br><br>**METHOD**<br>**Country:** Germany<br><br>**Participants**<br>**Recruitment:** From an outpatient clinic for patients with chronic pain<br>**Age:** Chronic pain: Mean 44.9. Depression: Mean 36.3. Controls: Mean 33.3<br>**Sex:** Chronic pain: 12 F, 8 M. Depression: 6 F, 16M. Controls: 21 F, 12 M

		**Total number of participants:** 75 (22 depression, 20 chronic low back pain, 33 controls)<br>**Dropouts:** 0<br>**Reason for drop out:** Not applicable<br>**Revised sample size:** As above<br>**Analgesia intake:** None in the healthy control group. Medication for depression and chronic pain in experiment group.<br>**Chronic conditions:** Controls: psychiatric conditions and chronic pain part of exclusion criteria. Depression group & chronic back pain group: no other psychiatric disorders as part of exclusion criteria<br>**Restrictions:** None stated<br><br>**<u>Study design:</u>**<br>Measurement of cortisol before and after heat pain in 3 groups (1) healthy taking no analgesia, (2) chronic pain (3) depression<br>**Other measurements:** Pain intensity<br>**Interventions:** Acute heat pain induced using the peltier device<br>**Comparison:** Change from baseline compared between the three groups<br>**Salivary assay:** Radioimmunoassay<br>**Saliva type:** Unclear<br>**Salivary collection method:** Swab<br><br>**<u>OUTCOMES</u>**<br>No statistically significant change in cortisol levels observed in any of the three groups<br>**Correlation with pain ratings:** No significant correlation<br>**Sex effects:** Not analysed
37	Nakajima (2011) Cortisol	**<u>AIM</u>**<br>To examine the extent to which pain perception prior to smoking cessation predicts early relapse<br><br>**<u>METHOD</u>**<br>**Country:** United States of America<br><br>**<u>Participants</u>**<br>**Recruitment:** Newspaper advertisements in the community and flyers in the university and participants completed a phone interview<br>**Age:** Mean in abstinent group 36.7. Mean in relapsed group 35.4<br>**Sex:** 46 F, 45 M<br>**Total number of participants:** 91<br>**Dropouts:** 0 for cortisol-CPT, 20 through the rest of the study<br>**Reason for drop out:** Follow up non-attendance<br>**Revised sample size:** 91 (all participants underwent CPT with saliva samples collected at the outset; dropout occurred later in the study)<br>**Analgesia intake:** None<br>**Chronic conditions:** Healthy (psychiatric disorders part of exclusion

		criteria, chronic pain not specifically reported)<br>**Restrictions:** Alcohol, smoking<br><br><u>**Study design:**</u><br>Salivary cortisol measured before and after an induced noxious cold stimulus prior to smoking cessation<br>**Other measurements:** Heart rate, blood pressure, pain intensity, pain characteristics, withdrawal symptoms, mood states, smoking status<br>**Interventions:** Acute cold pain induced using CPT<br>**Comparison:** Change from baseline. Changes compared between smokers who remained abstinent and smokers who relapsed<br>**Salivary assay:** Time-resolved fluorescence immunoassay with a cortisol-biotin conjugate as a tracer<br>**Saliva type:** Stimulated<br>**Saliva collection method:** Swab<br><br><u>**OUTCOMES**</u><br>Increase in salivary cortisol after CPT with no difference between the 2 groups<br>**Correlation with pain ratings:** Not analysed<br>**Sex effects:** Not analysed<br><br><u>**NOTES**</u><br>**Inclusion in the cortisol- cold pain quantitative analysis:** Yes<br><br><u>**BIAS**</u><br>**Bias Type:** High in confounding. Moderate in selection of participants, departures from intended exposure and selection of reported results.<br>**Author's judgement:** High ROB<br>**Support for judgement:** No control and unclear if steps were taken to minimise participant stress. Unclear whether all potential participants had equitable opportunity to be included. Interactions between participants and experimenters not fully described. No published pre-specified protocol.
38	Nelson (2001) Melatonin	<u>**AIM**</u><br>To examine the response of salivary melatonin to acute pain stimuli (electric stimulation)<br><br><u>**METHOD**</u><br>**Country:** United States of America<br><br><u>**Participants**</u><br>**Recruitment:** Details not reported<br>**Age:** Mean not reported (range: 19-55 years)<br>**Sex:** 7 F, 11 M<br>**Total number of participants:** 18

		**Dropouts:** 0<br>**Reason for drop out:** Not applicable<br>**Revised sample size:** 18<br>**Analgesia intake:** None<br>**Chronic conditions:** Healthy (psychiatric conditions not specifically reported, chronic pain part of exclusion criteria)<br>**Restrictions:** None reported<br><br>**<u>Study design</u>**<br>Salivary melatonin measured before and after an induced noxious stimulus<br>**Other measurements:** Pain intensity<br>**Intervention:** Acute pain induced by electric stimulation<br>**Salivary Assay:** Salivary melatonin by direct radioimmunoassay<br>**Comparison:** Change from baseline<br>**Saliva type:** Unstimulated saliva<br>**Saliva collection method:** Not recorded<br><br>**<u>OUTCOMES</u>**<br>Melatonin levels changed less than 5 minutes after the pain stimulus with initial decrease followed by a rise and then a reduction until levels similar to those anticipated for the time of day reached<br>**Correlation with pain ratings:** Not analysed<br>**Sex effects:** Not analysed
39	Niedbala (2018) Cortisol	**<u>AIM</u>**<br>To determine whether retaliating against a threatening outgroup, enables individuals in a group endure more pain and actually feel less pain intensity<br><br>**<u>METHOD</u>**<br>**Country:** United States of America<br><br>**<u>Participants</u>**<br>**Recruitment:** Introductory psychology students (no further details)<br>**Age:** Mean 19.32<br>**Sex:** 48 F**,** 26 M<br>**Total number of participants:** 74<br>**Dropouts:** 0<br>**Reason for dropouts:** Not applicable<br>**Revised sample size:** 74<br>**Analgesia intake:** Not reported<br>**Chronic conditions:** Healthy (psychiatric conditions and chronic pain not specifically reported)<br>**Restrictions:** Smoking, caffeine, mouth rinse before commencement<br><br>**<u>Study design:</u>**

		Salivary cortisol measured before and after an induced noxious cold stimulus compared between retaliation and non-retaliation groups, where completing CPT was a way of subtracting points from the rival (i.e. positive appraisal of pain)<br>**Other measurements:** Skin conductance, anger, approach motivation, pain intensity<br>**Intervention:** Acute cold pain induced using CPT<br>**Comparison**: Change from baseline. Change compared between different behaviour manipulation groups.<br>**Salivary assay:** Commercially available enzyme immunoassay<br>**Saliva type:** Stimulated<br>**Salivary collection method:** Passive drool<br><br>**<u>OUTCOMES</u>**<br>The cortisol response was inhibited in participants with positive appraisal of pain compared with controls, even though they did not report less pain.<br>**Correlation with pain ratings:** Not analysed<br>**Sex effects:** Not analysed<br><br>**<u>NOTES</u>**<br>**Inclusion in the cortisol- cold pain quantitative analysis:** No<br>**Reason:** Experimental design included emotional manipulation or a cognitive task as well as CPT
40	Olsson (2011)<br>Cortisol	**<u>AIM</u>**<br>To investigate the differences in physiologic and subjective parameters between lying on a bed of nails compared to a soft bed, and, whether there are any differences between listening to relaxation instructions on a CD versus no CD-instructions<br><br>**<u>METHOD</u>**<br>**Country:** Sweden<br><br>**<u>Participants</u>**<br>**Recruitment:** Internet advertisements and by posters at work places near the study location<br>**Age:** Mean 39.7<br>**Sex:** 20 F, 12 M<br>**Total number of participants:** 32<br>**Dropouts:** 3<br>**Reason for drop out:** Saliva samples turned up dry to the lab and could not be analyzed<br>**Revised sample size:** 29<br>**Analgesia intake:** Not reported<br>**Chronic conditions:** Healthy (psychiatric conditions and chronic pain not specifically recorded)<br>**Restrictions:** Caffeine, nicotine and any medication 12 hours prior to

		participation<br><br>**Study design:**<br>Salivary cortisol measured before and after a noxious mechanical stimulus compared with a soft stimulus<br>**Other measurements:** End-tidal carbon dioxide, oxygen saturation, respiration rate, heart rate, heart rate variability, skin conductance level, blood pressure, ECG, pain intensity<br>**Intervention:** Acute pain induced by mechanical pressure pain (lying on a Shakti-mat), compared with lying on a soft bed, both with and without listening to a relaxing music CD<br>**Comparison:** Change from baseline, compared in 4 groups: A—lying on nails in silence, B—lying on nails with CD at comfortable volume, C—lying on soft bed in silence, D—lying on a soft bed listening to CD<br>**Salivary assay:** Spectria [125I]-Coated Tube Radioimmunoassay<br>**Saliva type:** Stimulated<br>**Salivary collection method:** Swab<br><br>**OUTCOMES**<br>No effects of either bed type or relaxing instructions on saliva cortisol<br>**Correlation with pain ratings:** No correlation; no rise though participants reporting rapid and significant rise in pain at the start of lying on nail bed<br>**Sex effects:** Not analysed<br><br>**NOTES**<br>Healthy participants habituated to the induced pain on the nail bed and were able to subjectively relax. When on the nail bed, signs of both sympathetic and parasympathetic nervous system activity were observed.
41	Quartana (2010) Cortisol	**AIM**<br>To examine the relationship between trait pain catastrophizing and morning salivary cortisol levels before and after pain induction in pain free and temporomandibular disorder (TMD) participants and whether TMD patients had greater hyperalgesia and hypercortisolism<br><br>**METHOD**<br>**Country:** United States of America<br><br>**Participants**<br>**Recruitment:** TMD patients: from a dental school-based, orofacial pain clinic and media advertisements for a larger prospective study concerning sleep disturbance and TMD pain and function. Healthy controls: from fliers posted at a major teaching hospital and medical school<br>**Age:** Mean: TMD 33.79 and controls 25.91<br>**Sex:** TMD: 32 F, 7 M. Healthy: 21 F, 1 M<br>**Total number of participants:** 39 TMD, 22 healthy controls<br>**Dropouts:** 0

		**Reason for drop out:** Not applicable<br>**Revised sample size:** 61<br>**Analgesia intake:** None<br>**Chronic conditions:** Healthy or TMD (psychiatric conditions part of exclusion criteria, chronic pain excluded in healthy group<br>**Restrictions:** Smoking, food, caffeine, exercise<br><br>**Study design:**<br>Salivary cortisol measured before and after noxious cold, pressure and heat stimuli in TMD and control participants<br>**Other measurements:** Pain catastrophizing, psychological distress, pain intensity<br>**Interventions:** Acute pain induced by a combination of pressure, heat and cold stimuli induced using pressure algometry at defined anatomical sites, peltier stimulator, CPT<br>**Comparison:** Change from baseline compared between TMD and healthy participants<br>**Salivary assays:** Commercially available enzyme immunoassay (EIA)<br>**Saliva type:** Unstimulated<br>**Salivary collection method:** Swab<br><br>**OUTCOMES**<br>No difference in cortisol response from baseline to post-pain between the people with TMD and healthy people. In a separate analysis of the same experiment, in people who had a tendency to catastrophizing there was a reduction in salivary cortisol immediately and 20 minutes after pain compared to baseline<br>**Correlation with pain ratings:** No significant correlation<br>**Sex effects:** Not analysed<br><br>**NOTES**<br>**Inclusion in cortisol-cold analysis:** No<br>**Reason:** Pain induced by a combination of noxious stimuli with no separate analysis of cold pain
43	Schneider (2022) Cortisol Alpha-amylase	**AIM**<br>To evaluate the effect of psychosocial stress (Trier Social Stress Test, TSST) combined with performance feedback on changes in pain perception and their association with neuroendocrine stress parameters<br><br>**METHOD**<br>**Country:** Germany<br><br>**Participants**<br>**Recruitment:** A web based software for recruiting participants (SONA Systems), postings in a university department of psychology, advertisements in a local on-line newspaper

		**Age:** 23.83<br>**Sex:** F only<br>**Total number of participants:** 186<br>**Dropouts:** 5<br>**Reason for drop out:** Experiment was uncomfortable or did not meet exclusion criteria (5), profound high cortisol levels (2), unaccounted for (2)<br>**Revised sample size:** 177<br>**Analgesia intake:** None<br>**Chronic conditions:** Healthy (psychiatric disorders and chronic pain part of exclusion criteria)<br>**Restrictions:** Smoking, food, alcohol, caffeine<br><br>**<u>Study design:</u>**<br>Salivary cortisol and alpha- amylase measured before and after induced noxious heat combined with one of: TSST followed by positive feedback (43), negative feedback (46) or no feedback (45) or TSST placebo version (43)<br>**Other measurements:** pain intensity, anxiety<br>**Interventions:** Acute phasic heat pain induced by thermal stimulator, tonic heat pain (water bath)<br>**Comparison:** Change after psychosocial stress induction and differences between the 3 study groups and placebo<br>**Salivary assay:** Cortisol: chemi-luminescence immunoassay with high sensitivity. Alpha-amylase: enzyme kinetic method<br>**Saliva type:** Not stated<br>**Salivary Collection method:** swab<br><br>**<u>OUTCOMES</u>**<br>In the group who received TSST placebo (n=43), cortisol levels dropped during the course of the experiment with a significant drop after the first experimental heat pain. There was no change in alpha amylase.<br>There was a rise in both biomolecules in response to TSST induced stress<br>**Correlation with pain ratings:** No correlation<br>**Sex effects:** Not applicable (female only study)<br><br>**<u>NOTES</u>**<br>Experimentally induced social stress did not influence pain in women with or without performance feedback.
42	Serrano (2019)<br>Alpha-amylase<br>Cortisol	**<u>AIM</u>**<br>To examine the association between the catechol-O-methyltransferase (COMT) allele and perceived pain, anxiety, cortisol and sAA levels<br><br>**<u>METHOD</u>**<br>**Country:** United States of America<br><br>**<u>Participants</u>**

		**Recruitment:** Flyers distributed on university campus<br>**Age:** 21.12 years<br>**Sex:** 45 F, 41 M<br>**Total number of participants:** 86<br>**Dropouts:** 0<br>**Reason for drop out:** Not applicable<br>**Revised sample size:** 86<br>**Analgesia intake:** Not reported<br>**Chronic conditions:** Healthy (psychiatric conditions and chronic pain not specifically reported)<br>**Restrictions:** None reported<br><br>**<u>Study design:</u>**<br>Salivary cortisol and sAA measured before and after an induced noxious cold stimulus<br>**Other measurements**: Anxiety, pain intensity<br>**Interventions:** Acute cold pain induced by CPT<br>**Comparison:** Change from baseline before and after CPT, compared between COMT Met allele carriers and Val homozygotes<br>**Salivary assay:** Cortisol: Human cortisol enzyme immunoassay (EIA). sAA: Kinetic Enzyme Assay<br>**Saliva type:** Unstimulated<br>**Collection method:** Passive drool<br><br>**<u>OUTCOMES</u>**<br>Significantly greater change in sAA in COMT Met allele carriers compared with Val homozygotes at the 20 minute post-CPT time point<br>**Correlation with pain ratings:** Not analysed<br>**Sex effects:** Not analysed<br><br>**<u>NOTES</u>**<br>Pain ratings increased significantly immediately after CPT but were not affected by COMT polymorphism. The authors concluded that the COMT genotype influences the stress response to painful stimuli.<br>**Inclusion in the cortisol- cold pain quantitative analysis:** Yes<br><br>**<u>BIAS</u>**<br>**Bias Type:** High in confounding. Moderate in selection of participants, departures from intended exposure and selection of the reported result.<br>**Author's judgement:** High ROB<br>**Support for judgement:** No control and unclear if steps were taken to minimise participant stress. Recruitment limited to university campus. Interactions between participants and experimenters not fully described. No published pre-specified protocol.
47	Smith-Hanrahan	**<u>AIM:</u>**<br>To examine change in salivary kallikriens in association with the stress

	(1997)<br>Kallikreins	response to abdominal surgery<br><br>**METHOD**<br>**Country:** Canada<br><br>**Participants**<br>**Recruitment:** Patients scheduled for surgery at Montreal General Hospital. No further details.<br>**Age:** Mean 43.9<br>**Sex:** Mixed, distribution not recorded<br>**Total number of participants:** 19<br>**Dropouts:** 3<br>**Reason for drop out:** Insufficient data<br>**Revised sample size:** 16<br>**Analgesia intake:** Not reported<br>**Chronic conditions:** Healthy (psychiatric conditions not specifically reported and chronic pain excluded)<br>**Restrictions:** Food, alcohol<br><br>**Study design**<br>Salivary kallikreins measured before and after gynaecological surgery<br>**Other measurements:** Plasma cortisol, pain intensity<br>**Intervention:** Elective hysterectomy with or without oophorectomy for benign disease<br>**Salivary Assay:** ELISA<br>**Comparison**: Change from baseline<br>**Saliva type:** Stimulated saliva<br>**Salivary collection method:** Swab<br><br>**OUTCOMES**<br>Kallikreins increased significantly at 2, 4, and 6 hours after surgery, but not at 1 hour<br>Peak increase in kallikreins was at the 4 hour time point (8x higher than pre-operative levels)<br>**Correlation with pain ratings:** Reported pain levels did not follow the pattern of change in kallikreins (pain intensity peaked at one hour and declined after this point)<br>**Sex effects:** Not analysed
49	Sobas (2020)<br>sTNFαR-II<br>sIgA<br>Alpha-amylase<br>Testosterone<br>Cortisol	**AIM**<br>To evaluate change in pain biomarkers in the saliva following Advanced Surface Ablation eye surgery, in order to determine their validity as objective pain measures<br><br>**METHOD**<br>**Country:** Spain

		**Participants**<br>**Recruitment:** Consecutive patients listed for corneal surface ablation surgery from ophthalmology clinic<br>**Age:** 28.78 ± 6.93<br>**Sex:** F 13, M 19<br>**Total number of participants:** 32<br>**Dropouts:** 0<br>**Reason for drop out:** Not applicable<br>**Revised sample size:** 32<br>**Analgesia intake:** None<br>**Chronic conditions:** Healthy (psychiatric conditions not reported, chronic pain part of exclusion criteria)<br>**Restrictions:** None reported<br><br>**Study design:**<br>Salivary sTNFαR-II, sIgA, sAA, testosterone & cortisol measured before and after eye surgery<br>**Comparison:** Change from pre-operative levels<br>**Interventions:** Acute pain after corneal surgery<br>**Salivary assays:** Cortisol: DRG® Salivary Cortisol ELISA, DRG® Instruments GmbH, Marburg, Germany.). Testosterone (DRG®Salivary Testosterone ELISA, DRG Instruments GmbH, Marburg, Germany). sAA (DRG Salivary Alpha Amylase ELISA, DRG Instruments GmbH, Marburg, Germany). sTNFαRII (Quantikine®, Human sTNF RII/TNFRSF1B Immunoassay, R&D Systems, Minneapolis, MN, USA). sIgA (Salimetrics® Salivary Secretory IgA ELISA, Pennsylvania, USA)<br>**Saliva type:** Stimulated saliva<br>**Collection method:** Passive drool<br><br>**OUTCOMES**<br>IgA: Significant increase one hour after surgery<br>sTNFαR-II: Significant reduction one hour after surgery<br>Cortisol: Rise in the immediate pre-operative period compared to baseline with a further rise 1 hour after surgery<br>sAA: No significant rise in sAA<br>Testosterone: No significant change<br>**Correlation with pain ratings:** IgA: Significant positive correlation one hour after surgery**.** sTNFαR-II: No correlation**.** Cortisol: No correlation<br>**Sex effects:** Not analysed
50	Tanaka (2021) Alpha-amylase	**AIM**<br>To compare the effect of Yokucansan (YKS), a traditional Japanese herbal (Kampo) medicine, vs placebo treatment on women undergoing breast cancer surgery without axillary clearance<br><br>**METHOD**<br>**Country:** Japan

		**<u>Participants</u>**<br>**Recruitment:** Not specified<br>**Age:** range 20-60<br>**Sex:** F only<br>**Total number of participants:** 100<br>**Dropouts:** 23<br>**Reason for drop out:** Protocol violation (1), refused participation (2), positive sentinel node (12), steroid treatment needed (1), incomplete data (7)<br>**Revised sample size:** 77 (35 YKS, 42 control)<br>**Analgesia intake:** None<br>**Chronic conditions:** Healthy (psychiatric conditions and chronic pain part of exclusion criteria)<br>**Restrictions:** none reported<br><br>**<u>Study design:</u>**<br>Salivary alpha amylase measured before and after breast surgery in women given YKS or placebo in a single blind randomised controlled trial<br>**Other measurements:** pain intensity, anxiety, depression, quality of life<br>**Interventions:** Breast surgery<br>**Comparison:** Change from pre-operative baseline within each group and also compared between the active treatment and placebo group<br>**Salivary assay:** Handheld monitor (COCORO meter, NIPRO, Osaka, Japan) with disposable test strip- assessment if reaction time of a hydrolyzing reaction<br>**Saliva type:** Unclear<br>**Salivary Collection method:** test strip<br><br>**<u>OUTCOMES</u>**<br>No change in salivary alpha-amylase in the different time points in the control group. In the YKS group, salivary alpha-amylase scores directly before operation were significantly lower than those on the day before the surgery and one day postoperatively<br>**Correlation with pain ratings:** Not analysed<br>**Sex effects:** Not applicable
44	Wittwer (2016) Alpha-amylase	**<u>AIM</u>**<br>To investigate the effects of acute heat pain on salivary alpha amylase activity<br><br>**<u>METHOD</u>**<br>**Country:** Switzerland<br><br>**<u>Participants</u>**<br>**Recruitment:** Not stated<br>**Age:** Mean 26

		**Sex:** 13 F, 14 M<br>**Total number of participants:** 27<br>**Dropouts:** 4<br>**Reason for drop out:** Unavailable pain intensity data (2), took analgesics (2)<br>**Revised sample size:** 23<br>**Analgesia intake:** None<br>**Chronic conditions:** Healthy (psychiatric conditions and chronic pain not specifically reported)<br>**Restrictions:** Caffeine, alcohol, food, precautions to avoid blood contamination<br><br>**<u>Study design:</u>**<br>Salivary AA measured before and after induced noxious heat stimulus<br>**Other measurements:** Pain intensity, mood and anxiety<br>**Intervention:** Acute heat pain induced using Medoc TSA-II thermode<br>**Comparison:** Change from baseline<br>**Salivary assay:** Alpha amylase enzyme activity using reagents<br>**Saliva type:** Unstimulated saliva<br>**Collection method:** Swab<br><br>**<u>OUTCOME</u>**<br>Significant rise in sAA after heat pain<br>**Correlation with pain ratings:** Positive correlation between sAA activity and pain intensity<br>**Sex effects:** No correlation<br>**<u>NOTES</u>**<br>No significant correlation found between the trait anxiety score and the pre-test sAA level<br>After the pain assessment, irrespective of gender, participants felt significantly calmer and their mood was better
51	Yamaguchi (2006) Alpha-amylase	**<u>AIM</u>**<br>To validate the use of salivary amylase activity, as an indicator of pain in people with severe disability who required the daily replacement of gastric and/or bronchial tubes<br><br>**<u>METHOD</u>**<br>**Country:** Japan<br><br>**<u>Participants</u>**<br>**Recruitment:** From a hospital setting, no further details<br>**Age:** Mean 20.5<br>**Sex:** 3 F, 7 M<br>**Total number of participants:** 10<br>**Dropouts:** 0<br>**Reason for drop out:** Not applicable

		**Revised sample size:** 10<br>**Analgesia intake:** Not reported<br>**Chronic conditions:** Severe motor and cognitive disabilities (psychiatric conditions and chronic pain not specifically reported)<br>**Restrictions:** None reported<br><br>**<u>Study design:</u>**<br>Salivary AA measured before and after a noxious medical procedure stimulus<br>**Other measurements:** Pain intensity, heart rate<br>**Intervention:** Acute pain after gastric or bronchial tube exchange<br>**Salivary assay:** Enzymatic reagent method<br>**Saliva type:** Stimulated<br>**Collection method:** Monitor device with test strip under the tongue<br><br>**<u>OUTCOMES</u>**<br>Significant rise in sAA<br>**Correlation with pain ratings:** Significant positive correlation found<br>**Sex effects:** Not analysed
45	Youssef (2018) Alpha-amylase	**<u>AIM</u>**<br>To investigate differences in cardiovascular biomarkers between different cold stimulus responder types<br><br>**<u>METHODS</u>**<br>**Country:** Canada<br><br>**<u>Participants</u>**<br>**Recruitment:** By social media, e-mail, and informational flyers<br>**Age:** Mean 24.7<br>**Sex:** 16 F, 16 M<br>**Total number of participants:** 32<br>**Dropouts:** 2 (both male)<br>**Reason for drop out:** Test not completed due to pain (2 M)<br>**Revised sample size:** 30<br>**Analgesia intake:** Not reported<br>**Chronic conditions:** Healthy (psychiatric conditions and chronic pain not specifically reported)<br>**Restrictions:** Caffeine, exercise, alcohol, food<br><br>**<u>Study design:</u>**<br>Salivary AA measured before and after an induced noxious cold stimulus<br>**Other measurements:** Measures of cardiovascular sympathetic tone (blood pressure, heart rate, cardiac output, stroke volume, left ventricular ejection timee, pre-ejection period), pain intensity<br>**Intervention:** Cold pain induced by CPT<br>**Comparison:** Change from baseline

		**Salivary assays:** sAA kinetic reaction immunoassay<br>**Saliva type:** Stimulated saliva<br>**Collection method:** Swab<br>**Restrictions:** All of food, alcohol, smoking, caffeine<br><br>**OUTCOMES**<br>Significant rise in sAA after cold pain<br>**Correlation with pain ratings:** Not analysed<br>**Sex effects:** Not analysed
46	Zimmer (2003) Cortisol	**AIM**<br>To examine sex differences in subjective pain and cortisol response to a noxious stimulus<br><br>**METHOD**<br>**Country:** Germany<br><br>**Participants**<br>**Recruitment:** University students (no other details)<br>**Age:** 22.32<br>**Gender:** 42 F, 42 M<br>**Total number of participants:** 84<br>**Dropouts:** 8<br>**Reasons for drop out:** Multivariate outliers (2 F, 3 M), strong autonomic reactions to noxious stimulus (3 F)<br>**Revised sample size:** 76 (37 F, 39 M)<br>**Analgesia intake:** Not reported<br>**Chronic conditions:** Healthy (psychiatric conditions and chronic pain not specifically reported)<br>**Restrictions:** Smoking<br><br>**Study design:**<br>Salivary cortisol measured before and after an induced noxious cold stimulus<br>**Other measurements:** Pain intensity & unpleasantness, anxiety, distress, blood pressure, heart rate<br>**Comparison:** Change from baseline. Comparison of change between the sexes<br>**Intervention:** Cold pain (plunge test)<br>**Salivary assay:** ELISA<br>**Saliva type:** Unstimulated<br>**Salivary collection method:** Swab<br><br>**OUTCOMES**<br>Salivary cortisol increased in both men and women, greater increase in men<br>**Correlation with pain ratings:** Significant positive correlation

		**Sex effects:** Significantly greater increase in salivary cortisol from baseline in men at 20 minutes after plunge test<br><br>**NOTES**<br>**Inclusion in the cortisol- cold pain quantitative analysis:** Yes<br><br>**BIAS**<br>**Bias Type:** Moderate in confounding, selection of participants, departures from intended exposure, missing data and selection of reported result<br>**Author's judgement:** Moderate ROB<br>**Support for judgement:** No control but steps taken to minimise participant stress. Recruitment limited to university students. Interactions between participants and experimenters not fully described. Missing data not included in analysis. No published pre-specified protocol.
